# The reporting quality of randomized controlled trials in Chinese herbal medicine (CHM) formulas for diabetes based on the consort statement and its extension for CHM formulas

**DOI:** 10.3389/fphar.2024.1288479

**Published:** 2024-01-22

**Authors:** Yan Liu, Chaoyue Hu, Kehua Zhou, Ye Zhang, Jing Kang, Yalu Wen, Ruyue Yuan, Jiaoyue Li, Qiyao Zhao, Li Zhang, Xiaohui Yang

**Affiliations:** ^1^ Beijing University of Chinese Medicine, Beijing, China; ^2^ Key Laboratory of Chinese Internal Medicine of Ministry of Education, Dongzhimen Hospital, Beijing University of Chinese Medicine, Beijing, China; ^3^ Dongzhimen Hospital, Beijing University of Chinese Medicine, Beijing, China; ^4^ Department of Hospital Medicine, ThedaCare Regional Medical Center-Appleton, Appleton, WI, United States; ^5^ Dongfang Hospital, Beijing University of Chinese Medicine, Beijing, China

**Keywords:** consort statement, Chinese herbal medicine formula, randomized controlled trials, diabetes, quality of reporting

## Abstract

**Background:** This study aimed to assess the overall reporting quality of randomized controlled trials (RCTs) in Chinese herbal medicine (CHM) formulas for patients with diabetes, and to identify factors associated with better reporting quality.

**Methods:** Four databases including PubMed, Embase, Cochrane Library and Web of Science were systematically searched from their inception to December 2022. The reporting quality was assessed based on the Consolidated Standards of Reporting Trials (CONSORT) statement and its CHM formula extension. The overall CONSORT and its CHM formula extension scores were calculated and expressed as proportions separately. We also analyzed the pre-specified study characteristics and performed exploratory regressions to determine their associations with the reporting quality.

**Results:** Seventy-two RCTs were included. Overall reporting quality (mean adherence) were 53.56% and 45.71% on the CONSORT statement and its CHM formula extension, respectively. The strongest associations with reporting quality based on the CONSORT statement were multiple centers and larger author numbers. Compliance with the CHM formula extension, particularly regarding the disclosure of the targeted traditional Chinese medicine (TCM) pattern (s), was generally insufficient.

**Conclusion:** The reporting quality of RCTs in CHM formulas for diabetes remains unsatisfactory, and the adherence to the CHM formula extension is even poorer. In order to ensure transparent and standardized reporting of RCTs, it is essential to advocate for or even mandate adherence of the CONSORT statement and its CHM formula extension when reporting trials in CHM formulas for diabetes by both authors and editors.

## 1 Introduction

Diabetes mellitus (DM), a chronic metabolic disorder, has become a major public health burden globally. The International Diabetes Federation (IDF) estimates that over 780 million people will be living with diabetes worldwide by 2045 (Sun et al., 2021). Diabetes and its complications not only cause severe detrimental health impacts for the individuals affected but also impose a significant socioeconomic burden on healthcare systems. Effective management of diabetes is crucial to mitigate the negative impacts on health, decrease the risks for complications, and improve the quality of life for those living with the disease.

Chinese herbal medicine (CHM) has been employed for centuries in the treatment and management of various ailments, including diabetes. CHM may improve serum glucose regulation (Yang et al., 2023). For example, ginseng was shown to positively impact glucose metabolism, reduce insulin resistance, and increase energy expenditure (Zhao et al., 2023).Cinnamon was found to have anti-inflammatory effects and the ability to lower fasting blood sugar levels (Liu et al., 2023a). A CHM formula is made of multiple botanical drugs with specific properties that, when combined, may enhance the overall therapeutic effects (Lee et al., 2022). Recent research indicates that CHM formulas may be beneficial in improving glycemic control and mitigating diabetes-related complications (Zhang L. et al., 2019). Examples of such formulas include some well-known Chinese patent medicines such as Jinlida granules (Pan et al., 2021), Liuwei Dihuang Pills (Shi et al., 2019), and Shenqi Jiangtang Granules (Tang et al., 2021), which are commonly employed for the purpose of regulating serum glucose levels by physicians in China. The use of CHM formulas as a complementary or alternative treatment for diabetes is common and wide spread in the Chinese healthcare system (Joeliantina et al., 2019). Yet, evidence to support their use in diabetes remains unknown and likely limited.

Randomized controlled trials (RCTs) are considered the gold standard for evaluating the efficacy and safety of medical interventions (Sibbald and Roland, 1998). High-quality RCTs are essential for providing reliable evidence on the effectiveness of CHM formulas (if any) in diabetes management. However, poor reporting quality of RCTs hinders the reproducibility, transparency, and interpretability of the research findings, ultimately affecting the clinical application and evidence synthesis of these interventions (Glasziou et al., 2014). The Consolidated Standards of Reporting Trials (CONSORT) Statement, introduced in 1996 and updated in 2010, is an evidence-based guideline aimed at improving the reporting quality of RCTs (Schulz et al., 2010). The CONSORT Statement consists of a 25-item checklist addressing various aspects of trial design, analysis, and interpretation. Additionally, an extension to the CONSORT Statement for CHM formulas (the CHM formula extension) was developed in 2017 to address the unique features and challenges associated with reporting trials of CHM interventions (Cheng et al., 2017). This extension encompasses 7 additional items specific to CHM formulas, focusing on aspects such as intervention details, quality control, and standardization.

Despite the availability of these reporting guidelines, the reporting quality of RCTs related to CHM formulas for diabetes remains unclear. The objective of this study was to systematically review and evaluate the reporting quality of RCTs in CHM formulas for diabetes based on the CONSORT Statement and its CHM formula extension. Furthermore, this study also aimed to identify factors associated with higher reporting quality, providing insights for researchers, clinicians, and policymakers to improve the design, execution, and reporting of future RCTs in this field.

## 2 Methods

### 2.1 Study design

This methodological study focused on the reporting quality of RCTs in CHM formulas for patients with diabetes. All procedures and reporting followed the Preferred Reporting Items for Systematic Review and Meta-Analyses (PRISMA) guidelines (Moher et al., 2009). This study was registered on PROSPERO (CRD42023400810), http://www.crd.york.ac.uk/PROSPERO.

### 2.2 Data sources

Four databases including PubMed, EMBASE, Web of Science, and the Cochrane Library were systematically searched from the inception of the databases to 31 Dec 2022. Relevant studies were retrieved using Medical Subject Heading terms or keywords combined with free text words, such as Chinese herbal medicine, traditional Chinese medicine, diabetes, diabetic and randomized controlled trials. These keywords were modified according to the requirements of different databases. The detailed search strategies are available in Appendix 1.

### 2.3 Eligibility criteria

#### 2.3.1 Inclusion criteria

Studies which met all the following criteria were included: (1) patients diagnosed with diabetes mellitus. (2) parallel group randomized controlled trials. (3) the intervention measures of the experimental group were CHM Formulas, CHM Formulas combined with Chemical drugs or CHM Formulas combined with non-pharmacological treatments.

#### 2.3.2 Exclusion criteria

Studies which met one of the following criteria was excluded: (1) randomized crossover trials. (2) RCTs that were presented at conferences or in brief reports like comments or letters. (3) study protocols. (4) articles not in English. (5) full-text paper not retrievable.

### 2.4 Study selection and data collection

Two reviewers conducted an initial screening of the search results based on the title and abstract of each RCT identified in the systematic review. Potentially eligible studies were selected for full-text screening. Disagreements were resolved by a third reviewer. The two reviewers individually extracted the following data from each included RCT: type of journal, year of publication, sample size, number of authors, research center(s), details of diabetes diagnoses, patterns based on traditional Chinese medicine syndrome differentiation principles (abbreviated as TCM patterns), and positivity (beneficial results) of primary outcome(s). Using a predetermined data extraction form, data extraction was carried out by skilled and pretrained reviewers, and quality control procedures were frequently carried out to guarantee the accuracy and consistency.

### 2.5 Quality of reporting assessment

The 25-item CONSORT statement (2017) and its 7-item CHM formula extension were used to evaluate the quality of reporting of the included RCTs. We scored each item 1 point for fully reporting, 0.5 point for partially reporting, and 0 point for not reporting. In addition, we answered “not applicable” for the items not necessary to report. The two reviewers (Y.L, C. H.) independently evaluated the included studies. The disagreements between the two reviewers were adjudicated by a third reviewer (J.K.). Then, for each reporting, we separately standardized the CONSORT and its CHM formula extension scores to percentages, excluding non-applicable items.

### 2.6 Standard evolution of traditional Chinese medicine

To enhance the accuracy and reproducibility of this study, we adhered to The ConPhyMP consensus for reporting CHM formulas (Heinrich et al., 2022). The scientific nomenclature of botanical drug components was standardized using Rivera et al. (Rivera et al., 2014) as a reference. Furthermore, validation was carried out using the “Plant of the World Online” (http://www.plantsoftheworldonline.org) and “The World Flora Online” (WFO, http://www.worldfloraonline.org/) databases.

### 2.7 Statistical analysis

Descriptive statistics were used to summarize the data. Univariate analyses were performed to explore the relationship between each factor and the dependent variable (i.e., CONSORT score or CHM extension score) using the chi-square test or Fisher’s exact test as appropriate. Simple linear regression was used to estimate the relationship between the dependent variable and publication year.

We also conducted multivariate regressions and prespecified subgroup analyses (Liu et al., 2023b) to investigate factors that influence the quality of reporting. These factors included: (1) type of journal (integrative and complementary medicine *versus* non-integrative and complementary medicine journals, definition according to Journal Citation Reports, https://jcr.clarivate.com/jcr/browse-journals), (2) year of publication (2009 and earlier *versus* 2010 and later for the CONSORT statement; 2016 and earlier *versus* 2017 and later for the CHM formula extension), (3) sample size (below median *versus* above median), (4) number of authors (below median *versus* above median), (5) multicenter (yes *versus* no), (6) type 2 diabetes mellitus (T2DM) studied only (yes *versus* no), (7) TCM patterns studied (yes *versus* no), and (8) positivity of primary outcome (yes *versus* no). To assess the multicollinearity among the predictor variables in the multivariable regression model, the variance inflation factor (VIF) was calculated for each variable. A VIF greater than 5 is generally considered to indicate problematic multicollinearity.

All the graphical representations and statistical analyses were conducted using R 4.2.3 (R Foundation for Statistical Computing, Vienna, Austria) with a 2-sided *p*-value of less than 0.05 considered significant.

## 3 Results

### 3.1 Characteristics of RCTs included

**FIGURE 1:**
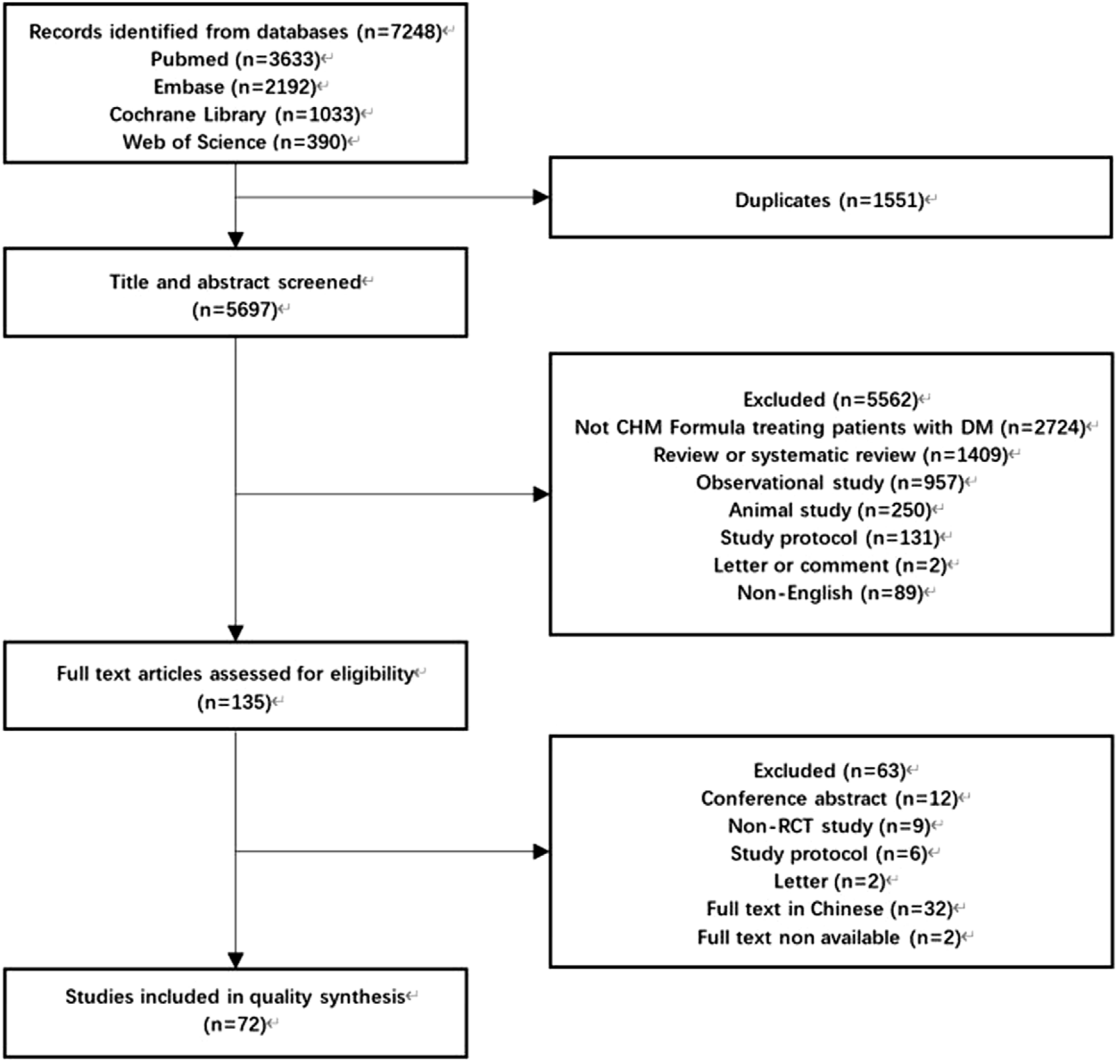
Flowchart of the literature screening process.

After initial screening, 7248 studies were obtained. After removing duplicates, we screened the titles and abstracts of 5697 articles and were successful in retrieving the full text of 135 articles, resulting in the inclusion of 72 articles (Hale et al., 1989; Zhu et al., 1992; Wang et al., 1997; Gao Y. et al., 1998; Li and Wang, 1999; Li et al., 2000; Ren, 2000; Wu et al., 2000; Nagaki et al., 2003; Huang et al., 2004; Chen and Wang, 2005; Wang and Wang, 2005; Xue et al., 2006; Wei and Xie, 2007; Leung et al., 2008; Zhang et al., 2008; Chao et al., 2009; Luo et al., 2009; You et al., 2009; Li S. et al., 2011; Li F. et al., 2011; Chen et al., 2012; Leung et al., 2012; Fang et al., 2013; Grant et al., 2013; Ji et al., 2013; Ma et al., 2013; Tong et al., 2013; Tsai et al., 2013; Tu et al., 2013; Ko et al., 2014; Watanabe et al., 2014; Xu et al., 2014; Zhao et al., 2014; Lian et al., 2015a; Lian et al., 2015b; Li et al., 2015; Liu H. et al., 2015; Guangcan and Ligong, 2015; Luo et al., 2015; Qiang et al., 2015; Zhang et al., 2015; Hu et al., 2016a; Hu et al., 2016b; Chui et al., 2016; Mo et al., 2016; Qiang et al., 2016; Wu et al., 2016; Yang et al., 2016; Zhao et al., 2016; Tian et al., 2017; Liu et al., 2018; Tang et al., 2018; Tong et al., 2018; Xiao et al., 2018; Yu et al., 2018; Zhao et al., 2018; Shi et al., 2019; Zhang Y. et al., 2019; Cui et al., 2019; Huang et al., 2019; Tassadaq and Wahid, 2019; Liu et al., 2020; Pan et al., 2021; Tang et al., 2021; Zhan et al., 2021; Liu et al., 2022; Lu et al., 2022; Qiao et al., 2022; Shi et al., 2022; Zhang et al., 2022; Zhu et al., 2022) that met the criteria (Figure 1). Among these studies, 24 (33.33%) RCTs studied T2DM (Hale et al., 1989; Huang et al., 2004; Chen and Wang, 2005; Chao et al., 2009; Chen et al., 2012; Grant et al., 2013; Ji et al., 2013; Tong et al., 2013; Tu et al., 2013; Watanabe et al., 2014; Xu et al., 2014; Lian et al., 2015b; Zhang et al., 2015; Hu et al., 2016a; Hu et al., 2016b; Chui et al., 2016; Qiang et al., 2016; Tian et al., 2017; Tang et al., 2018; Zhao et al., 2018; Zhang Y. et al., 2019; Huang et al., 2019; Pan et al., 2021; Zhang et al., 2022); 1 (1.39%) studied gestational diabetes mellitus (Tang et al., 2021); 35 (48.61%) studied diabetic complications (Wang et al., 1997; Gao Y et al., 1998; Li and Wang, 1999; Ren, 2000; Wu et al., 2000; Nagaki et al., 2003; Wei and Xie, 2007; Leung et al., 2008; Luo et al., 2009; You et al., 2009; Li S. et al., 2011; Li F. et al., 2011; Leung et al., 2012; Fang et al., 2013; Ma et al., 2013; Tsai et al., 2013; Ko et al., 2014; Lian et al., 2015a; Liu H. et al., 2015; Li et al., 2015; Luo et al., 2015; Mo et al., 2016; Yang et al., 2016; Zhao et al., 2016; Xiao et al., 2018; Cui et al., 2019; Shi et al., 2019; Tassadaq and Wahid, 2019; Liu et al., 2020; Zhan et al., 2021; Liu et al., 2022; Lu et al., 2022; Qiao et al., 2022; Shi et al., 2022; Zhu et al., 2022), with diabetic nephropathy being the most common (15 of them); and 12 (16.67%) studied diabetes with other comorbidities (Zhu et al., 1992; Li et al., 2000; Wang and Wang, 2005; Xue et al., 2006; Zhang et al., 2008; Zhao et al., 2014; Guangcan and Ligong, 2015; Qiang et al., 2015; Wu et al., 2016; Liu et al., 2018; Tong et al., 2018; Yu et al., 2018). Only 16 (22.22%) RCTs reported TCM patterns (Zhu et al., 1992; Gao Y et al., 1998; Li et al., 2000; Wu et al., 2000; Huang et al., 2004; Luo et al., 2009; You et al., 2009; Zhao et al., 2014; Guangcan and Ligong, 2015; Li et al., 2015; Wu et al., 2016; Yang et al., 2016; Tong et al., 2018; Xiao et al., 2018; Yu et al., 2018; Qiao et al., 2022). The main reported TCM patterns were blood stasis (*n* = 10), and qi deficiency (*n* = 8). The distribution details of types of diabetes and its complications studied were presented in Appendix 2.

The included RCTs were published from 1989 to 2022, of which 39 (54.17%) RCTs were published in complementary alternative medicine journals (Zhu et al., 1992; Wang et al., 1997; Gao Y et al., 1998; Li and Wang, 1999; Li et al., 2000; Ren, 2000; Wu et al., 2000; Nagaki et al., 2003; Huang et al., 2004; Chen and Wang, 2005; Xue et al., 2006; Zhang et al., 2008; Luo et al., 2009; You et al., 2009; Li S. et al., 2011; Li F. et al., 2011; Chen et al., 2012; Grant et al., 2013; Ma et al., 2013; Tsai et al., 2013; Tu et al., 2013; Watanabe et al., 2014; Zhao et al., 2014; Lian et al., 2015a; Guangcan and Ligong, 2015; Luo et al., 2015; Qiang et al., 2015; Hu et al., 2016b; Chui et al., 2016; Mo et al., 2016; Qiang et al., 2016; Yang et al., 2016; Zhao et al., 2016; Liu et al., 2018; Zhao et al., 2018; Zhan et al., 2021; Liu et al., 2022; Zhang et al., 2022; Zhu et al., 2022) and 33 (45.83%) RCTs were published in non-integrative and complementary medicine journals (Hale et al., 1989; Wang and Wang, 2005; Wei and Xie, 2007; Leung et al., 2008; Chao et al., 2009; Leung et al., 2012; Fang et al., 2013; Ji et al., 2013; Tong et al., 2013; Ko et al., 2014; Xu et al., 2014; Liu H. et al., 2015; Lian et al., 2015b; Li et al., 2015; Zhang et al., 2015; Hu et al., 2016a; Wu et al., 2016; Tian et al., 2017; Tang et al., 2018; Tong et al., 2018; Xiao et al., 2018; Yu et al., 2018; Zhang Y. et al., 2019; Cui et al., 2019; Huang et al., 2019; Shi et al., 2019; Tassadaq and Wahid, 2019; Liu et al., 2020; Pan et al., 2021; Tang et al., 2021; Lu et al., 2022; Qiao et al., 2022; Shi et al., 2022); 30 (41.67%) RCTs were multicenter trials (Wang and Wang, 2005; Wei and Xie, 2007; Leung et al., 2008; Chao et al., 2009; Luo et al., 2009; Li S. et al., 2011; Li F. et al., 2011; Leung et al., 2012; Grant et al., 2013; Ji et al., 2013; Tong et al., 2013; Tu et al., 2013; Watanabe et al., 2014; Xu et al., 2014; Lian et al., 2015a; Lian et al., 2015b; Li et al., 2015; Hu et al., 2016a; Hu et al., 2016b; Chui et al., 2016; Mo et al., 2016; Qiang et al., 2016; Yang et al., 2016; Tian et al., 2017; Tang et al., 2018; Tong et al., 2018; Yu et al., 2018; Shi et al., 2019; Liu et al., 2020; Liu et al., 2022) *versus* 42 (58.33%) RCTs were single center trials (Hale et al., 1989; Zhu et al., 1992; Wang et al., 1997; Gao Y et al., 1998; Li and Wang, 1999; Li et al., 2000; Ren, 2000; Wu et al., 2000; Nagaki et al., 2003; Huang et al., 2004; Chen and Wang, 2005; Xue et al., 2006; Zhang et al., 2008; You et al., 2009; Chen et al., 2012; Fang et al., 2013; Ma et al., 2013; Tsai et al., 2013; Ko et al., 2014; Zhao et al., 2014; Liu H. et al., 2015; Guangcan and Ligong, 2015; Luo et al., 2015; Qiang et al., 2015; Zhang et al., 2015; Wu et al., 2016; Zhao et al., 2016; Liu et al., 2018; Xiao et al., 2018; Zhao et al., 2018; Zhang Y. et al., 2019; Cui et al., 2019; Huang et al., 2019; Tassadaq and Wahid, 2019; Pan et al., 2021; Tang et al., 2021; Zhan et al., 2021; Lu et al., 2022; Qiao et al., 2022; Shi et al., 2022; Zhang et al., 2022; Zhu et al., 2022); 58 (80.56%) RCTs were positive (Zhu et al., 1992; Wang et al., 1997; Gao Y. et al., 1998; Li and Wang, 1999; Li et al., 2000; Ren, 2000; Wu et al., 2000; Nagaki et al., 2003; Huang et al., 2004; Chen and Wang, 2005; Wang and Wang, 2005; Xue et al., 2006; Wei and Xie, 2007; Leung et al., 2008; Zhang et al., 2008; Chao et al., 2009; Luo et al., 2009; You et al., 2009; Li S. et al., 2011; Li F.et al., 2011; Chen et al., 2012; Leung et al., 2012; Fang et al., 2013; Ji et al., 2013; Ma et al., 2013; Tong et al., 2013; Tsai et al., 2013; Xu et al., 2014; Zhao et al., 2014; Lian et al., 2015a; Lian et al., 2015b; Liu H. et al., 2015; Guangcan and Ligong, 2015; Qiang et al., 2015; Zhang et al., 2015; Hu et al., 2016a; Hu et al., 2016b; Chui et al., 2016; Mo et al., 2016; Wu et al., 2016; Yang et al., 2016; Tian et al., 2017; Liu et al., 2018; Xiao et al., 2018; Zhao et al., 2018; Shi et al., 2019; Zhang Y. et al., 2019; Cui et al., 2019; Huang et al., 2019; Tassadaq and Wahid, 2019; Liu et al., 2020; Pan et al., 2021; Tang et al., 2021; Zhan et al., 2021; Liu et al., 2022; Qiao et al., 2022; Shi et al., 2022; Zhu et al., 2022) *versus* 14 (19.44%) RCTs were negative (Hale et al., 1989; Grant et al., 2013; Tu et al., 2013; Ko et al., 2014; Watanabe et al., 2014; Li et al., 2015; Luo et al., 2015; Qiang et al., 2016; Zhao et al., 2016; Tang et al., 2018; Tong et al., 2018; Yu et al., 2018; Lu et al., 2022; Zhang et al., 2022) for the primary outcome. In addition, the median sample size was 95 (IQR 62–149.25) and the median number of authors was 6 (IQR 4–10.75) in the included RCTs. Additional characteristics for the included RCTs were presented in Table 1.

**TABLE 1:** Characteristics of included randomized controlled trials.

Characteristic	All (N = 72)/n (%)
Research on T2DM only ^a^	
Yes	24 (33.33%)
No	48 (66.67%)
Integrative and Complementary Medicine journals ^b^	
Yes	39 (54.17%)
No	33 (45.83%)
Number of Authors ^c^	
≤6	38 (52.78%)
>6	34 (47.22%)
Sample Size ^c^	
≤95	36 (50.00%)
>95	36 (50.00%)
Multicenter	
Yes	30 (41.67%)
No	42 (58.33%)
Patterns Studied ^d^	
Yes	16 (22.22%)
No	56 (77.78%)
Positivity of Primary Outcome ^e^	
Yes	58 (80.56%)
No	14 (19.44%)

^a^ RCTs, that exclusively studied type 2 diabetes mellitus were classified as ‘yes’, while those that studied gestational diabetes mellitus, diabetic complications, or other comorbidities were classified as ‘no’.

^b^ The classification of integrative and complementary medicine journals was based on the categorization from Journal Citation Reports. (https://jcr.clarivate.com/jcr/browse-journals).

^c^ The classification was according to median. The median of number of authors was 6. The median of sample size was 95.

^d^ The report explicitly stated that the TCM, pattern for the trial was classified as ‘yes’; otherwise, the classification was ‘no’.

^e^ The ‘yes’ classification indicated that the primary outcome had a *p*-value less than or equal to 0.05, while a ‘no’ classification indicated that the primary outcome had a *p*-value greater than 0.05.

### 3.2 Description of CHM formula

This study included 63 traditional Chinese herbal formulas, such as Liuwei Dihuang Pills, Jinlida, Compound Danshen Dripping Pill, etc. An examination of the unique flavors within each Chinese herbal formula revealed a total of 114 distinct types of Chinese botanical drugs, with five emerging as the most widely favored, including -as shown in “scientific plant name” [family; synonyms] (86): Rehmannia glutinosa (Gaertn.) DC. [Orobanchaceae; radix rehmanniae praeparata] (38.89%), Astragalus mongholicus Bunge. [Fabaceae; astragali radix] (38.89%), Salvia miltiorrhiza Bunge. [Lamiaceae; radix salviae miltiorrhizae] (26.39%), Cornus officinalis Siebold & Zucc. [Cornaceae; corni fructus] (25%), Dioscorea oppositifolia L. [Dioscoreaceae; dioscoreae rhizoma] (22.22%). Chinese medicinal preparations boast a diverse array of dosage forms, spanning both oral and topical administrations. We found that most included reports did not mention the quality control. The composition and characteristics of CHM formula were listed in Appendix 3.

### 3.3 Reporting quality based on the CONSORT statement

**FIGURE 2:**
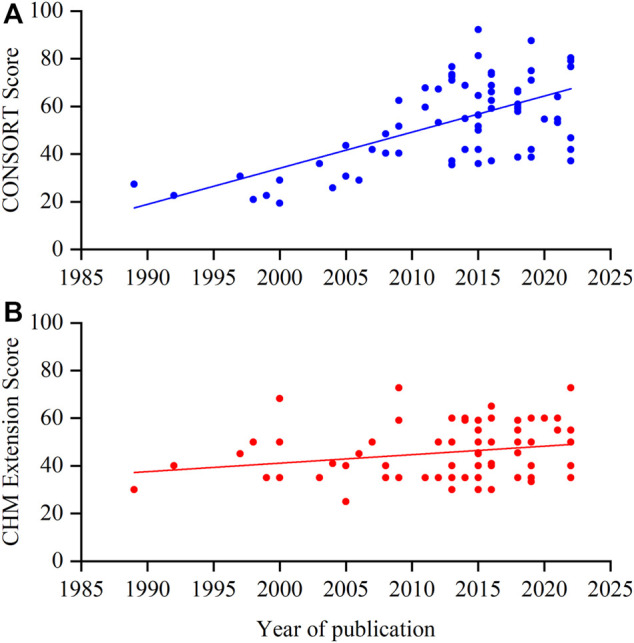
Scatterplot of the overall score per year. **(A)** CONSORT score; **(B)** CHM Formula extension score.

The mean adherence of the included RCTs to the CONSORT statement was 53.56% (SD 17.98%, min-max 19.35%–92.19%): 44 (61.11%) RCTs had adherence below 60% (Tang et al., 2021; Zhu et al., 2022; Shi et al., 2022; Qiao et al., 2022; Zhan et al., 2021; Liu et al., 2020; Tassadaq and Wahid, 2019; Cui et al., 2019; Zhao et al., 2018; Xiao et al., 2018; Tian et al., 2017; Tang et al., 2018; Zhao et al., 2016; Wu et al., 2016; Zhang et al., 2015; Xu et al., 2014; Guangcan and Ligong, 2015; Luo et al., 2015; Liu H. et al., 2015; Lian et al., 2015a; Zhao et al., 2014; Watanabe et al., 2014; Ma et al., 2013; Fang et al., 2013; Chen et al., 2012; Li F. et al., 2011; You et al., 2009; Luo et al., 2009; Zhang et al., 2008; Leung et al., 2008; Wei and Xie, 2007; Xue et al., 2006; Chen and Wang, 2005; Wang and Wang, 2005; Huang et al., 2004; Nagaki et al., 2003; Wu et al., 2000; Ren, 2000; Li et al., 2000; Li and Wang, 1999; Gao Y et al., 1998; Wang et al., 1997; Zhu et al., 1992), and only 4 (5.56%) had adherence above 80% (Lian et al., 2015b; Li et al., 2015; Huang et al., 2019; Lu et al., 2022). The reporting quality of the CONSORT statement tended to increase over time based on the publication year (Figure 2A).

**FIGURE 3:**
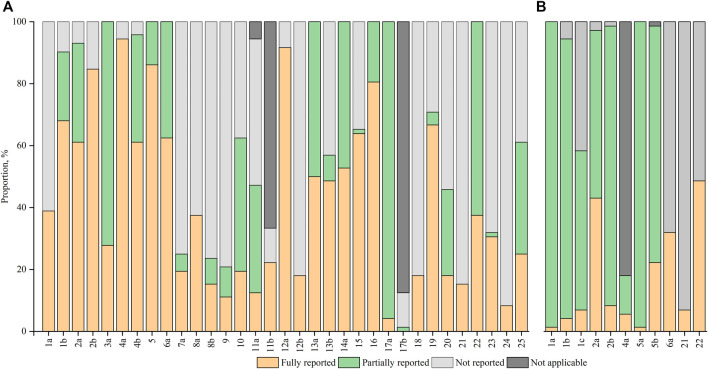
Proportion of reporting for individual items **(A)** CONSORT items; **(B)** CHM Formula extension items.

After excluding the non-applicable items, 11 items were fully reported in at least 60% of the included RCTs, of which 5 items were fully reported in more than 80% of the included RCTs. Surprisingly, less than 20% of the included RCTs reported all the methodological items of sample size (item 7a), randomization (items 8b, 9, and 10), blinding (item 11a), and statistical methods (item 12b). Figure 3A exhibited the percentages in reporting of the CONSORT items.

### 3.4 Reporting quality based on CHM formula extension

The mean CHM Formula extension reporting compliance for the included RCTs was 45.71% (SD 11.15%, min-max 25%–72.73%): 39 (54.17%) of these RCTs had an adherence rate lower than 50% (Hale et al., 1989; Zhu et al., 1992; Wang et al., 1997; Li and Wang, 1999; Ren, 2000; Nagaki et al., 2003; Huang et al., 2004; Chen and Wang, 2005; Wang and Wang, 2005; Xue et al., 2006; Leung et al., 2008; Zhang et al., 2008; Chao et al., 2009; Li S. et al., 2011; Li F. et al., 2011; Leung et al., 2012; Grant et al., 2013; Ji et al., 2013; Ma et al., 2013; Tong et al., 2013; Tsai et al., 2013; Ko et al., 2014; Xu et al., 2014; Lian et al., 2015a; Lian et al., 2015b; Li et al., 2015; Zhang et al., 2015; Hu et al., 2016a; Hu et al., 2016b; Wu et al., 2016; Yang et al., 2016; Tian et al., 2017; Liu et al., 2018; Tong et al., 2018; Cui et al., 2019; Shi et al., 2019; Tassadaq and Wahid, 2019; Lu et al., 2022; Zhang et al., 2022). The improving trend of reporting quality of the CHM formula extension over time was not as significant as the CONSORT statement (Figure 2B).

No item was adequately reported in more than 50% of the RCTs. Less than 10% of the RCTs reported all 7 items: title (1a), abstract (1b), keyword (1c), introduction (2b), participants (4a), interventions for CHM Formula (Lee et al., 2022), and generalizability (Lu et al., 2022). Among these included trials, 49 (68.06%) RCTs did not report outcome measures with TCM patterns (Shi et al., 2019; Zhu et al., 2022; Zhang et al., 2022; Shi et al., 2022; Lu et al., 2022; Zhang Y. et al., 2019; Tassadaq and Wahid, 2019; Cui et al., 2019; Zhao et al., 2018; Tong et al., 2018; Tian et al., 2017; Tang et al., 2018; Liu et al., 2018; Zhao et al., 2016; Yang et al., 2016; Wu et al., 2016; Hu et al., 2016a; Hu et al., 2016b; Qiang et al., 2016; Chui et al., 2016; Zhang et al., 2015; Xu et al., 2014; Qiang et al., 2015; Luo et al., 2015; Liu H. et al., 2015; Lian et al., 2015a; Lian et al., 2015b; Ko et al., 2014; Tu et al., 2013; Tsai et al., 2013; Tong et al., 2013; Ma et al., 2013; Grant et al., 2013; Leung et al., 2012; Chen et al., 2012; Li S. et al., 2011; Li F. et al., 2011; Chao et al., 2009; Zhang et al., 2008; Leung et al., 2008; Xue et al., 2006; Chen and Wang, 2005; Wang and Wang, 2005), while 67 (93.06%) RCTs did not explore the applicability of trial findings to different TCM patterns (Fang et al., 2013; Grant et al., 2013; Ji et al., 2013; Ma et al., 2013; Tong et al., 2013; Tsai et al., 2013; Tu et al., 2013; Ko et al., 2014; Watanabe et al., 2014; Xu et al., 2014; Zhao et al., 2014; Lian et al., 2015a; Liu H. et al., 2015; Lian et al., 2015b; Guangcan and Ligong, 2015; Li et al., 2015; Luo et al., 2015; Qiang et al., 2015; Zhang et al., 2015; Hu et al., 2016a; Hu et al., 2016b; Chui et al., 2016; Qiang et al., 2016; Wu et al., 2016; Yang et al., 2016; Tian et al., 2017; Liu et al., 2018; Tang et al., 2018; Tong et al., 2018; Xiao et al., 2018; Yu et al., 2018; Cui et al., 2019; Shi et al., 2019; Tassadaq and Wahid, 2019; Liu et al., 2020; Pan et al., 2021; Tang et al., 2021; Zhan et al., 2021; Liu et al., 2022; Lu et al., 2022; Qiao et al., 2022; Shi et al., 2022; Zhang et al., 2022; Zhu et al., 2022). The percentages of RCTs adherence to each CHM formula extension item were shown in Figure 3B.

### 3.5 Factors associated with reporting quality

**FIGURE 4:**
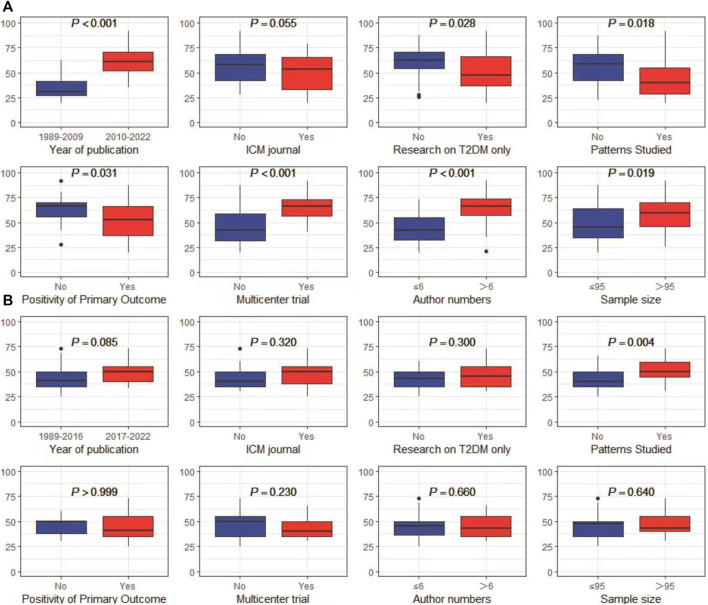
Results of univariate analyses **(A)** CONSORT score; **(B)** CHM Formula extension score.

In univariate analyses, the CONSORT scores were significantly different according to the predetermined factors. RCTs published more recently, conducted in multiple centers, RCTs with more authors and with a larger sample size had a better quality of reporting (refer to Figure 4A). RCTs reporting negative results exhibited superior quality compared to those reporting positive results. Furthermore, these findings suggested that the CONSORT statement score did not change in RCTs targeting specific TCM patterns. However, the CHM formula extension score was significantly higher in RCTs with the TCM patterns reported (refer to Figure 4B).

**FIGURE 5:**
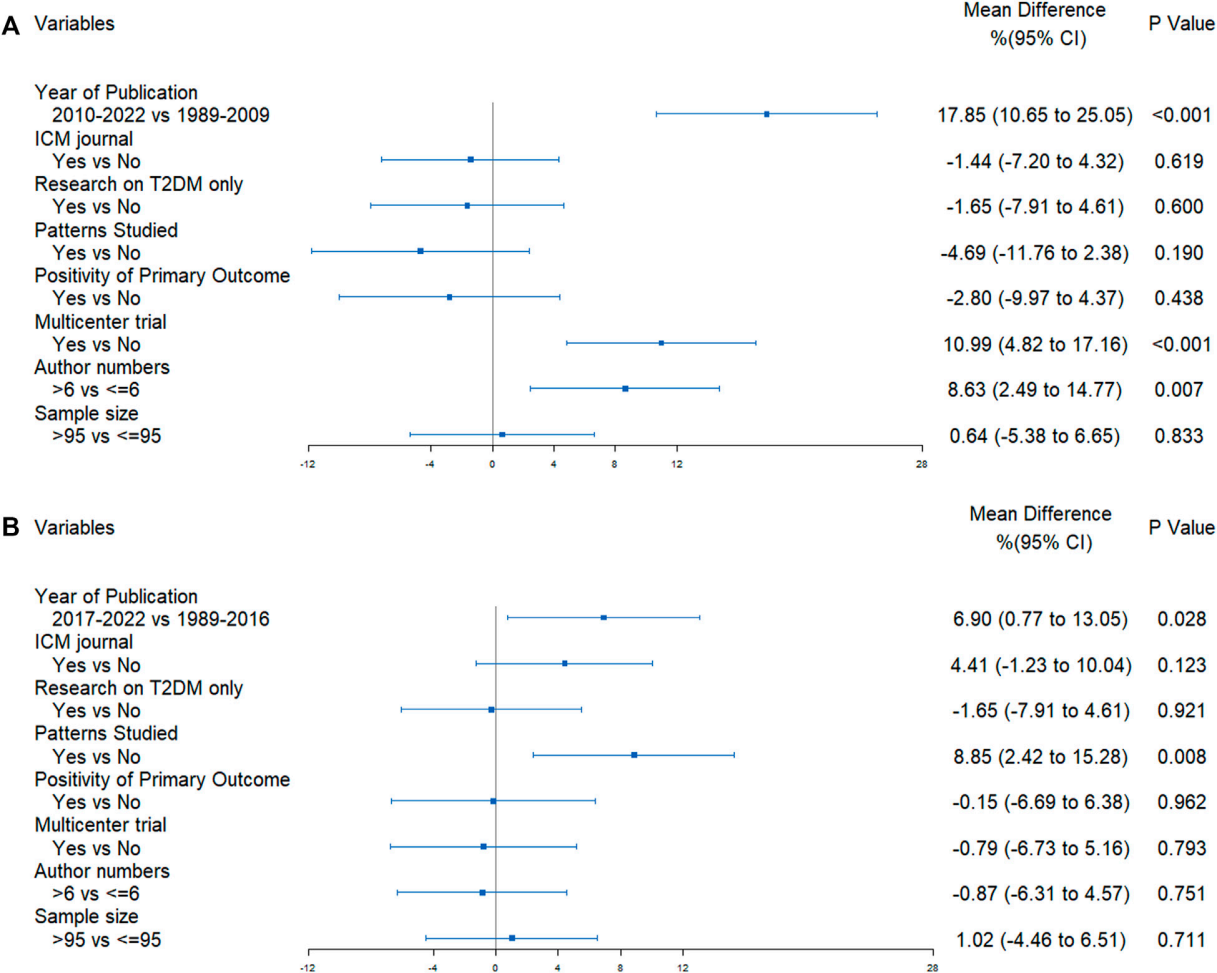
Forest plots of the results of multivariate regression analyses **(A)** CONSORT score; **(B)** CHM Formula extension score.

The results of multivariate regression analysis revealed that significant improvements in reporting quality following the implementation of the CONSORT (2010) statement, with an increase of 17.85% (95%CI 10.65%–25.05%, *p* < 0.001) in reporting quality. Multicenter studies demonstrated an improvement of 10.99% (95%CI 4.82%–17.16%, *p* < 0.001) in reporting quality when compared to single-center studies. Reports authored by more than six individuals displayed an 8.63% (95%CI 2.49%–14.77%, *p* = 0.007) improvement in quality (Figure 5A). Additionally, the publication of the CHM formula extension in 2017 yielded an improvement of 6.90% (95%CI 0.77%–13.05%, *p* = 0.028) in the quality of reporting, and an appreciable increase of 8.85% (95%CI 2.42%–15.28%, *p* = 0.008) was noted in the quality of reporting for TCM patterns in RCTs (Figure 5B).

## 4 Discussion

### 4.1 Principal findings

The overall quality of RCT reporting, as assessed by adherence to the CONSORT statement, demonstrated a general improvement over time, but the improvement in adherence to the CHM formula extension over time was not as significant as that to the CONSORT statement. Additionally, this study identified influential factors on reporting quality for the CONSORT statement, such as the publication year, the number of research centers, and the number of authors. For the CHM formula extension, the year of publication and the examination of TCM patterns were found to be significant factors affecting the reporting quality.

#### 4.1.1 CONSORT statement

In this study, reporting of RCTs in CHM formulas for patients with diabetes had a moderate level of adherence to the CONSORT statement, while compliance with the CHM formula extension was poor. The suboptimal reporting quality of RCTs in CHM formulas may be attributed to a lack of awareness and/or emphasis on the CONSORT statement (and its CHM formula extension) in the field of CHM research (Li et al., 2012; Ma et al., 2016a). It has been observed that most reviewers and editors in the field of CHM or complementary and integrative medicine did not require authors to adhere to the CONSORT statement when submitting reports and did not evaluate RCTs using these guidelines. Consequently, authors often provided insufficient descriptions of the essential information. Numerous studies have been identified with inadequate reporting of crucial details, particularly in the methods section, which may significantly impact the trustworthiness and reliability of the study results. In our investigation, the caliber of reports within the methodology section was inferior relative to other portions, with fewer than one-fifth of RCTs furnishing comprehensive accounts of sample size computations, randomization procedures, and the execution of blinding. This observation aligns with previous evaluations of other RCTs, namely, RCTs of diabetes, CHM formulas, and acupuncture (Liu X. et al., 2015; Zhai et al., 2015; Yang et al., 2022). Of particular concern, 54 RCTs in the present study did not report the calculations used to determine the sample size. This issue is worrisome because effect size represents subjective clinical judgment, and incorrect assumptions may introduce bias into the trial results (Schulz and Grimes, 2005; Wang et al., 2007). Without such information, readers cannot discern potential biases in the study design and researchers will not be able to replicate the findings.

Additionally, only a limited number of studies provided comprehensive information on randomization. Specifically, item 8b (type of randomization) and item 9 (allocation concealment mechanism) were frequently underreported, potentially reducing the authenticity of the RCTs’ results (Balshem et al., 2011; Savović et al., 2012). Many studies merely mentioned “random assignment” or “randomly” without elaborating on the method used for generating the random assignment sequence, whether allocation concealment was employed, or if the performers were separated. In this study, a mere nine RCTs provided an in-depth account on who was blinded after assignment to interventions and how. This deficiency suggests that some RCTs may not have implemented blinding, which could influence subject compliance with the intervention and the evaluation of outcome indicators by trialists, particularly for subjective outcome indicators (Wood et al., 2008; Hróbjartsson and Boutron, 2011). Consequently, the internal validity of the study findings may be compromised by these shortcomings. Only 12.5% of reports accurately illustrated the use of blinding procedures, compared to 57% reported in RCTs published in the journals of Diabetes Care, Diabetes and Diabetologia from 2011 to 2013 (Zhai et al., 2015), which indicated that the methodology reporting for RCTs of CHM formulas for diabetes remains to be improved.

The presentation of results in item 17a was found to be insufficient. Among the analyzed RCTs, only three reported results for each group, along with the estimated effect size and its precision. In contrast, the majority of studies merely provided descriptive statistics and *p*-values, neglecting to include effect values that represent between-group differences and confidence intervals that convey the precision of estimates. This inadequate presentation of outcomes renders their interpretation unclear, as statistical significance alone does not adequately capture clinical relevance (Gardner and Altman, 1986). Therefore, it is essential to require reports to furnish complete and accurate disclosure of results, ensuring proper assessment and interpretation of their clinical relevance.

#### 4.1.2 CHM formula extension

The CONSORT statement extension dictates that RCTs related to CHM formulas offer comprehensive reports on the diagnostic criteria of TCM patterns based on traditional Chinese medicine syndrome differentiation principles (Cheng et al., 2017). Unfortunately, a mere four RCTs adhered to this requirement and provided detailed diagnostic criteria, inclusion/exclusion criteria for subjects with TCM patterns, and appropriate reference sources in their reports. In contrast, most reports only referenced the Western medicine-defined disease targeted by the clinical trials, neglecting to mention the specific TCM pattern. Additionally, the CHM formula extension necessitates a thorough description of the botaniacal ingredients, quality certification, preparation methods, and prescription changes in the methods section (Cheng et al., 2017), ensuring the reproducibility of the intervention approach. Regrettably, only a single RCT provided a comprehensive report on all the required details associated with botanical ingredients.

#### 4.1.3 Factors associated with reporting quality

This study revealed that larger sample sizes were associated with better compliance to the CONSORT statement in univariate analyses. However, when the data was examined using multivariate regression analyses, the influence of sample size was found to be insignificant. The trials examining the effectiveness of CHM formulas in treating patients with diabetes revealed a median sample size of 95 (IQR 62–149.25). A statistical review of RCT reports from three leading diabetes journals indicated a median sample size of 103 (IQR 31–328) (Zhai et al., 2015), which showed the field of diabetes research has relatively few large-scale RCTs. The multivariate analysis detected a significant improvement in reporting quality following the introduction of both the CONSORT statement and its CHM formula extension. The previous research revealed that overall CONSORT score of RCTs in CHM formula published during 2010–2011 increased 15.30 (95%CI 8.34 to 22.26, *p* = 0.001) when compared to studies published during 2007–2008. It is evident that the awareness of CONSORT statement has grown over time (Liu X. et al., 2015). A similar trend was observed in the implementation of other CONSORT extensions (Ma et al., 2016b; Chhapola et al., 2016; Chen et al., 2022). Whether the primary outcome was positive had no impact on the reporting quality of the included RCTs. This might be explained by the fact that the majority of the results of trials for diabetes were positive (Zhai et al., 2015). Furthermore, this study disclosed that adherence to the CHM formula extension for reporting increased when relevant TCM patterns was addressed. Accurate TCM pattern differentiation is essential in guiding the prescription of CHM formulas (Lu et al., 2012). The finding in this study underscores that TCM pattern differentiation may not only improve patient outcome but also the reporting quality of RCTs in CHM formulas. The pivotal challenge in clinical research in TCM lies in implementing pattern differentiation in the design of clinical trials. As a method of patient stratification, TCM pattern differentiation holds the potential to transform clinical trial strategies and promote the development of superior clinical trials in CHM formulas (Jiang et al., 2012).

### 4.2 Comparison with other studies

The CONSORT statement has undergone numerous updates and expansions since its inception, with additional guidelines introduced for specific interventions such as nonpharmacologic treatments (Boutron et al., 2017) and acupuncture (MacPherson et al., 2010). These extensions have proven valuable in assessing the quality of relevant RCT reports within their respective domains (Ma et al., 2016b; Liu et al., 2021). However, it is noteworthy that despite the availability of the CHM formula extension, introduced in 2017, its utilization appears to be less widespread compared to other CONSORT extensions. The CHM formula extension offers tailored guidance for evaluating the methodological rigor and transparency of RCTs in CHM formulas. In our study, we acknowledge the existence of the CONSORT extension for CHM formulas and recognize its potential in improving the reporting quality of RCTs in CHM formulas. Additionally, we also highlight the observed variation in its use and emphasize the need for increased awareness and adoption within the research community.

The comprehensiveness of reporting is positively influenced by journal policies (Anders et al., 2021). To improve reporting quality, it is recommended that journals explicitly outline their requirements and expectations for authors to follow the CONSORT statement and its CHM formula extension in their submission guidelines. Advocating for the early application of these guidelines in the research process is essential. This strategic approach not only bolsters reporting quality but also lays the groundwork for methodological robustness, thereby reinforcing the overall credibility and integrity of study outcomes. To ensure a more inclusive perspective, we propose extending our considerations to journals in other languages, such as those in Japanese, which may report studies related to Kampo medicine. This broader approach is essential for fostering international collaboration, enhancing the global visibility of reporting standards, and facilitating cross-cultural dialogue within the scientific community.

Diabetes causes significant socioeconomic burden in the modern society despites the development of various new medications over the past decades. CHM formulas may provide a completely new field of diabetes management. Assessing the quality of RCTs in CHM formulas helps bridge the gap between diabase treatments using CHM formulas and the modern diabetes management. Our research contributes to public health in some significant ways. Firstly, by evaluating the reporting quality of RCTs in CHM formulas in diabetes management, our study may help provide a more rigorous understanding of CHM’s efficacy and safety. Secondly, in the modern era of evidence-based practice, our findings will help refine the quality of RCT in CHM formulas, aiding healthcare professionals in making informed decisions of CHM formulas based on the best available evidence (Aletaha et al., 2023). Thirdly, results of this study may provide policymakers and health professionals guidance in the development of research funding requirements, promoting the integration of effective and well-researched CHM treatments into modern healthcare.

### 4.3 Limitations of this study

This study has some limitations. Firstly, we only included published articles written in English. Secondly, our scoring system assigned a score of 1 to each item, without considering the varying importance of different items to the overall quality of an RCT report. Consequently, some items that were more critical may have been given equal weight as less critical items. Additionally, two RCT reports may receive identical scores even though the quality of specific sections of reporting may differ. This scoring approach could result in an oversimplified understanding of RCT’s reporting quality. The overall reporting ratio offers only an overview of the reporting quality without evaluating the quality of each part, making a direct comparison between different RCTs infeasible. Lastly, the reporting quality does not equal the quality of the included RCTs *per se*.

## 5 Conclusion

The CONSORT statement has been used in clinical research for over 2 decades; however, despite the growing improvements, compliance with the CONSORT statement in RCTs of CHM formulas for diabetes was suboptimal. Reporting quality seems to be higher for trials conducted by multiple centers and those involving a greater number of authors. In contrast, compliance with the CHM formula extension, particularly regarding the disclosure of the targeted TCM pattern (s), was generally insufficient. To improve the overall reporting quality of RCTs in CHM formulas, it is essential to promote adherence to the CHM formula extension by encouraging collaborative engagement among authors, journals, and practitioners.

## Data Availability

The original contributions presented in the study are included in the article/Supplementary material, further inquiries can be directed to the corresponding author.
