# Supplementary material for: The reporting quality of randomized controlled trials in Chinese herbal medicine (CHM) formulas for diabetes based on the consort statement and its extension for CHM formulas
Source: Front Pharmacol. 2024 Jan 22;15:1288479. doi: 10.3389/fphar.2024.1288479 (PMC10839105; doi:10.3389/fphar.2024.1288479)
Supplement: Supplementary file 1 [file DataSheet1.docx]

**Appendix 1 Search strategy**

PubMed

1 Medicine, Chinese traditional [MeSH Terms]

2 Herbal Medicine [MeSH Terms]

3 Traditional Medicine, Chinese OR Traditional Chinese Medicine OR Traditional Chinese Medicines OR Chinese Traditional Medicine OR Chinese medicines OR Chinese medicine OR Chinese Medicine, Traditional OR Drugs, Chinese Herbal OR Herb OR Herbs OR Herbal Medicines OR Medicine, Herbal OR Chinese Herbal Drugs OR Herbal Drugs, Chinese

4 #1 OR #2 OR #3

5 Diabetes Mellitus[MeSH Terms]

6 Diabetes OR Diabetic OR T1DM OR T2DM OR Type 1 Diabetes OR Type 2 Diabetes

7 #5 OR #6

8 (((((("Randomized Controlled Trial" [Publication Type]) OR "Controlled Clinical Trial" [Publication Type]) OR "Double Blind Method"[Mesh]) OR "Single-Blind Method "[Mesh]) OR "Prospective Studies"[Mesh])) OR "Comparative Study" [Publication Type]

9 "Randomized Controlled Trial"[tiab] OR "Controlled Clinical Trial"[tiab] OR "random allocation"[tiab] OR "double blind method"[tiab] OR "single-blind method"[tiab] OR "clinical trial"[tiab] OR "prospective studies"[tiab] OR "Double Blind"[tiab] OR "Double Masked"[tiab] OR "Single-Blind"[tiab] OR "Single Masked"[tiab] OR "Prospective Studies"[tiab] OR "Prospective Study"[tiab] OR "Randomized clinical trials"[tiab] OR "Randomized clinical trial"[tiab] OR "randomized trials"[tiab] OR "randomized trial"[tiab] OR ((random*[tiab] OR Control*[tiab]) AND trial*[tiab]) OR "Comparative Study"[tiab] OR "Comparative Studies"[tiab] OR "Comparative analysis"[tiab] OR Comparativ*[tiab] AND (analys*[tiab] OR effectiveness[tiab] OR benefit[tiab]) OR "placebo"[tiab]

10 #8 OR #9

11 ((("Humans"[Mesh]) OR "Patients"[Mesh]) OR "Men"[Mesh]) OR "Women"[Mesh]

12 human[tiab] OR patient*[tiab] OR man[tiab] OR women[tiab]

13 #11 OR #12

14 #4 AND #7 AND #10 AND #13

Web of Science

1. TS=(Medicine, Chinese traditional OR Herbal Medicine OR Traditional Medicine, Chinese OR Traditional Chinese Medicine OR Traditional Chinese Medicines OR Chinese Traditional Medicine OR Chinese medicines OR Chinese medicine OR Chinese Medicine, Traditional OR Drugs, Chinese Herbal OR Herb OR Herbs OR Herbal Medicines OR Medicine, Herbal OR Chinese Herbal Drugs OR Herbal Drugs, Chinese)
2. TS=(Diabetes Mellitus OR Diabetes OR Diabetic OR T1DM OR T2DM OR Type 1 Diabetes OR Type 2 Diabetes)
3. TS=("Randomized Controlled Trial" OR "Controlled Clinical Trial" OR "random allocation" OR "double blind method" OR "single-blind method" OR "prospective studies" OR "Double Blind" OR "Double Blind" OR "Double Masked" OR "Double Masked" OR "Single-Blind" OR "Single-Blind" OR "Single Masked" OR "Single Masked" OR "Prospective Studies" OR "Prospective Study" OR "Randomized clinical trials" OR "Randomized clinical trial" OR "randomized trials" OR "randomized trial" OR "Comparative Study" OR "Comparative Studies" OR "Comparative analysis" OR (Comparativ* AND (analys* OR effectiveness OR benefit)))
4. TI=((Randomized OR controlled OR blinded OR placebo) AND (trial OR trials OR study OR Studies OR analysis))
5. #3 OR #4
6. TS=(human OR patient* OR man OR women)
7. #1 AND #2 AND #5 AND #6

Cochrane Library

1 MeSH descriptor: [Medicine, Chinese Traditional] explode all trees

2 MeSH descriptor: [Herbal Medicine] explode all trees

3 Traditional Medicine, Chinese OR Traditional Chinese Medicine OR Traditional Chinese Medicines OR Chinese Traditional Medicine OR Chinese medicines OR Chinese medicine OR Chinese Medicine, Traditional OR Drugs, Chinese Herbal OR Herb OR Herbs OR Herbal Medicines OR Medicine, Herbal OR Chinese Herbal Drugs OR Herbal Drugs, Chinese

4 #1 OR #2 OR #3

5 MeSH descriptor: [Diabetes Mellitus] explode all trees

6 Diabetes OR Diabetic OR T1DM OR T2DM OR Type 1 Diabetes OR Type 2 Diabetes

7 #5 OR #6

8 MeSH descriptor: [Randomized Controlled Trial] this term only

9 MeSH descriptor: [Controlled Clinical Trial] this term only

10 MeSH descriptor: [Double-Blind Method] this term only

11 MeSH descriptor: [Single-Blind Method] this term only

12 MeSH descriptor: [Prospective Studies] this term only

13 MeSH descriptor: [Multicenter Study] this term only

14 MeSH descriptor: [Comparative Study] this term only

15 MeSH descriptor: [Random Allocation] this term only

16 MeSH descriptor: [Controlled Clinical Trials as Topic] this term only

17 "Randomized Controlled Trial" OR "Controlled Clinical Trial" OR "random allocation" OR "double blind method" OR "single-blind method" OR "prospective studies" OR "Double Blind" OR "Double Blind" OR "Double Masked" OR "Double Masked" OR "Single-Blind" OR "Single-Blind" OR "Single Masked" OR "Single Masked" OR "Prospective Studies" OR "Prospective Study" OR "Randomized clinical trials" OR "Randomized clinical trial" OR "randomized trials" OR "randomized trial" OR "Comparative Study" OR "Comparative Studies" OR "Comparative analysis" OR (Comparativ* AND (analys* OR effectiveness OR benefit))

18 (Randomized OR controlled OR blinded OR placebo) AND (trial OR trials OR study OR Studies OR analysis)

19 #8 OR #9 OR #10 OR #11 OR #12 OR #13 OR #14 OR #15 OR #16 OR #17 OR #18

20 MeSH descriptor: [Patients] explode all trees

21 MeSH descriptor: [Men] explode all trees

22 MeSH descriptor: [Women] explode all trees

23 MeSH descriptor: [Humans] explode all trees

24 #20 OR #21 OR #22 OR #23

25 #4 AND #7 AND #19 AND #24

Embase

1 'chinese medicine'/exp OR 'chinese medicine'

2 'herbal medicine'/exp OR 'herbal medicine'

3 ‘Medicine, Chinese traditional’ OR ‘Traditional Medicine, Chinese’ OR ‘Traditional Chinese Medicine’ OR ‘Traditional Chinese Medicines’ OR ‘Chinese Traditional Medicine’ OR ‘Chinese medicines’ OR ‘Chinese Medicine, Traditional’ OR ‘Drugs, Chinese Herbal’ OR ‘Herb’ OR ‘Herbs’ OR ‘Herbal Medicines’ OR ‘Medicine, Herbal’ OR ‘Chinese Herbal Drugs’ OR ‘Herbal Drugs, Chinese’

4 #1 OR #2 OR #3

5 'diabetes mellitus'/exp OR 'diabetes mellitus'

6 'Diabetes' OR 'Diabetic' OR 'T1DM' OR 'T2DM' OR 'Type 1 Diabetes' OR 'Type 2 Diabetes'

7 #5 OR #6

8 'randomized controlled trial'/de OR 'controlled clinical trial'/de OR 'double blind procedure'/de OR 'single-blind procedure'/de OR 'prospective study'/de OR 'multicenter study'/de OR 'comparative study'/de OR 'randomization'/de OR 'randomized controlled trial (topic)'/de OR 'controlled clinical trial (topic)'/de

1. 'human'/exp OR 'patient'/exp OR 'male'/exp OR 'female'/exp
2. #4 AND #7 AND #8 AND #9

**Appendix 2 The distribution of all included trials**

| Type of Diseases | All(N=72) / n(%) |
| --- | --- |
| Type 2 Diabetes Mellitus | **24 (33.33%)** |
| Gestational Diabetes Mellitus | **1 (1.39%)** |
| Diabetic Complications | **35 (48.61%)** |
| Diabetic Nephropathy | 15 (20.83%) |
| Diabetic Neuropathy | 5 (6.94%) |
| Diabetic Retinopathy | 4 (5.56%) |
| Diabetic Angiopathy | 2 (2.78%) |
| Diabetic Foot | 8 (11.11%) |
| Diabetic Gastroparesis | 1 (1.39%) |
| Comorbidities | **12 (16.67%)** |
| Metabolic Diseases | 5 (6.94%) |
| Hyperlipidemias | 5 (6.94%) |
| Obesity | 1 (1.39%) |
| Pulmonary Diseases | 1 (1.39%) |
| Pulmonary Tuberculosis | 1 (1.39%) |
| Cerebrovascular Diseases | 2 (2.78%) |
| Vascular Dementia | 1 (1.39%) |
| Acute Ischemic Stroke | 1 (1.39%) |
| Cardiac Diseases | 2 (2.78%) |
| Coronary Heart Disease | 1 (1.39%) |
| Myocardial Ischemia | 1 (1.39%) |
| Ophthalmic & Otorhinolaryngologic Diseases | 2 (2.78%) |
| Xerophthalmia | 1 (1.39%) |
| Hearing loss | 1 (1.39%) |

**Appendix 3 Description of CHM formula.**

| Reference | Name of CHM | Type of Formula | Source | Quality Control Reported | Composition of CHM |
| --- | --- | --- | --- | --- | --- |
| Zhu JH et al. (2022) | Shenkang injection | injection | Xi’an Century Shengkang Pharmaceutical Co., Ltd. | No | Rheum officinale Baill. [Polygonaceae; radix et rhizoma rhei], Astragalus mongholicus Bunge. [Fabaceae; astragali radix], Salvia miltiorrhiza Bunge. [Lamiaceae; radix salviae miltiorrhizae], Carthamus tinctorius L. [Asteraceae; carthami flos] |
| Zhang JJ et al. (2022) | TCM herbal tea | tea | Suzhou Tianling TCM Pieces Co., Ltd. | No | Astragalus mongholicus Bunge. [Fabaceae; astragali radix], Morus alba L. [Moraceae; mori fructus], Zea mays L. [Poaceae; amylum maydis], Ophiopogon japonicus (Thunb.) Ker Gawl. [Asparagaceae; ophiopogonis radix], Gynostemma pentaphyllum (Thunb.) Makino [Cucurbitaceae; gynostemmatis herba], Lycium barbarum L. [Solanaceae; lycii fructus] |
| Zhuang S et al. (2022) | Zhachong Shisanwei pills | pill | Inner Mongolia Datang Pharmaceutical Co..LTD.. | No | Aconitum kusnezoffii Rchb. [Ranunculaceae; aconiti kusnezoffii radix], Terminalia chebula Retz. [Combretaceae; chebulae fructus], Acorus gramineus Aiton [Acoraceae; acori gramineri rhizoma], Dolomiaea costus (Falc.) Kasana & A.K.Pandey [Asteraceae; aucklandiae radix], Syzygium aromaticum (L.) Merr. & L.M.Perry [Myrtaceae; caryophylli flos], Aquilaria sinensis (Lour.) Spreng. [Thymelaeaceae; aquilariae lignum resinatum], Glycyrrhiza glabra L. [Fabaceae; glycyrrhizae radix et rhizoma], Myristica fragrans Houtt. [Myristicaceae; myristicae semen], Moschus [not botanical drug], Acropora echinata [not botanical drug], Margarita [not botanical drug], Limonite [not botanical drug], Magnetite [not botanical drug]. |
| Qiao YL et al. (2022) | medicinal liquid application with Tangbiling herbs | liquid | Not Stated | No | Astragalus mongholicus Bunge. [Fabaceae; astragali radix], Glycyrrhiza glabra L. [Fabaceae; glycyrrhizae radix et rhizoma], Neolitsea cassia (L.) Kosterm. [Lauraceae; cinnamomi cortex], Paeonia lactiflora Pall. [Paeoniaceae; paeoniae radix alba], Angelica sinensis (Oliv.) Diels [Apiaceae; angelicae sinensis radix], Asarum heterotropoides F.Schmidt [Aristolochiaceae; asari radix et rhizoma], Tetrapanax papyrifer (Hook.) K.Koch [Araliaceae; medulla tetrapanacis], Lonicera japonica Thunb. [Caprifoliaceae; caulis lonicerae japonicae], Zingiber officinale Roscoe [Zingiberaceae; rhizoma zingiberis praeparatum], Ziziphus jujuba Mill. [Rhamnaceae; fructus jujubae] |
| Lu QY et al. (2022) | Xiaoketongbi Formula | granule | Guangdong Provincial Hospital of Chinese Medicine | Yes | Rheum officinale Baill. [Polygonaceae; radix et rhizoma rhei], Astragalus mongholicus Bunge. [Fabaceae; astragali radix], Salvia miltiorrhiza Bunge. [Lamiaceae; radix salviae miltiorrhizae], Angelica sinensis (Oliv.) Diels [Apiaceae; angelicae sinensis radix], Prunus persica (L.) Batsch [Rosaceae; persicae semen] |
| Liu J et al. (2022) | Zicuiyin decoction | decoction | Hebei Meiwei Pharmaceutical Co., Ltd. | Yes | Astragalus mongholicus Bunge. [Fabaceae; astragali radix], Rehmannia glutinosa (Gaertn.) DC. [Orobanchaceae; radix rehmanniae praeparata], Dioscorea oppositifolia L. [Dioscoreaceae; dioscoreae rhizoma], Cornus officinalis Siebold & Zucc. [Cornaceae; corni fructus] |
| Zhan HB et al. (2021) | Ruyi Jinhuang powder paste | powder | Beijing Tongrentang | No | Rheum officinale Baill. [Polygonaceae; radix et rhizoma rhei], Glycyrrhiza glabra L. [Fabaceae; glycyrrhizae radix et rhizoma], Curcuma longa L. [Zingiberaceae; curcumae longae rhizoma], Phellodendron amurense Rupr. [Rutaceae; phellodendri cortex pulveratus], Atractylodes lancea (Thunb.) DC. [Asteraceae; atractylodis lanceae rhizoma pulveratum], Magnolia officinalis Rehder & E.H.Wilson [Magnoliaceae; flos magnoliae officinalis], Citrus × aurantium f. deliciosa (Ten.) M.Hiroe [Rutaceae; citri exocarpium rubrum], Arisaema erubescens (Wall.) Schott [Araceae; arisaema cum bile], Angelica dahurica (Hoffm.) Benth. & Hook.f. ex Franch. & Sav. [Apiaceae; angelicae dahuricae radix], Trichosanthes kirilowii Maxim. [Cucurbitaceae; trichosanthis semen tostum] |
| Tang J et al. (2021) | Shenqi Jiangtang Granules | granule | Shandong Lunan Houpu Pharmaceutical Co., Ltd., | No | Astragalus mongholicus Bunge. [Fabaceae; astragali radix], Lycium barbarum L. [Solanaceae; lycii fructus], Ophiopogon japonicus (Thunb.) Ker Gawl. [Asparagaceae; ophiopogonis radix], Rehmannia glutinosa (Gaertn.) DC. [Orobanchaceae; radix rehmanniae praeparata], Panax ginseng C.A.Mey. [Araliaceae; folium ginseng], Alisma plantago-aquatica subsp. orientale (Sam.) Sam. [Alismataceae; alismatis rhizoma], Schisandra chinensis (Turcz.) Baill. [Schisandraceae; schisandrae chinensis fructus] |
| Pan JM et al. (2021) | Jinlida granules | granule | Not Stated | No | Panax ginseng C.A.Mey. [Araliaceae; folium ginseng], Atractylodes macrocephala Koidz. [Asteraceae; atractylodis rhizoma pulveratum], Poria cocos (Schw. ) Wolf. [Polyporaceae; Poria], Pueraria montana var. lobata (Willd.) Maesen & S.M.Almeida ex Sanjappa & Predeep [Fabaceae; radix puerariae lobatae], Polygonatum odoratum (Mill.) Druce [Asparagaceae; fragrant solomonseal rhizome] |
| Liu YL et al. (2020) | Cortex Phellodendri Compound Fluid | liquid | Shandong Hanfang Pharmaceutical Co. | No | Phellodendron amurense Rupr. [Rutaceae; phellodendri cortex pulveratus], Lonicera japonica Thunb. [Caprifoliaceae; caulis lonicerae japonicae], Forsythia suspensa (Thunb.) Vahl [Oleaceae; forsythiae fructus], Taraxacum mongolicum Hand.-Mazz. [Asteraceae; radix cum herba taraxaci], Scolopendra [not botanical drug] |
| Zhang Y et al. (2019) | Shenqi compound particle | partical | Chinese Pharmacy of the Hospital of Chengdu University of TCM | No | Panax ginseng C.A.Mey. [Araliaceae; folium ginseng], Astragalus mongholicus Bunge. [Fabaceae; astragali radix], Dioscorea oppositifolia L. [Dioscoreaceae; dioscoreae rhizoma], Cornus officinalis Siebold & Zucc. [Cornaceae; corni fructus], Rehmannia glutinosa (Gaertn.) DC. [Orobanchaceae; radix rehmanniae praeparata], Salvia miltiorrhiza Bunge. [Lamiaceae; radix salviae miltiorrhizae], Trichosanthes kirilowii Maxim. [Cucurbitaceae; trichosanthis semen tostum], Rheum officinale Baill. [Polygonaceae; radix et rhizoma rhei] |
| Tassadaq,N. (2019) | Tricardin(danshenform 250mg dripping pills capsules) | capsule | Not Stated | No | Not Stated |
| Shi RF et al. (2019) | Liuwei Dihuang Pills | pill | Wanxi Pharmaceutical Co., Ltd. | No | Not Stated |
| Huang YH et al. (2019) | YH1 | granule | Sun Ten Pharmaceutical Co., Ltd. | Yes | Glycyrrhiza glabra L. [Fabaceae; glycyrrhizae radix et rhizoma], Ziziphus jujuba Mill. [Rhamnaceae; fructus jujubae], Dioscorea oppositifolia L. [Dioscoreaceae; dioscoreae rhizoma], Panax ginseng C.A.Mey. [Araliaceae; folium ginseng], Atractylodes macrocephala Koidz. [Asteraceae; atractylodis rhizoma pulveratum], Poria cocos (Schw. ) Wolf. [Polyporaceae; Poria], Coptis chinensis Franch. [Ranunculaceae; coptidis rhizoma], Lablab purpureus subsp. purpureus [Fabaceae; semen lablab album], Nelumbo nucifera Gaertn. [Nelumbonaceae; folium nelumbinis], Platycodon grandiflorus (Jacq.) A.DC. [Campanulaceae; platycodi radix], Coix lacryma-jobi var. ma-yuen (Rom.Caill.) Stapf [Poaceae; coicis semen], Wurfbainia villosa (Lour.) Škorničk. & A.D.Poulsen [Zingiberaceae; amomi fructus] |
| Cui FQ et al. (2019) | Baoshenfang Formula | decoction | Not Stated | Yes | Astragalus mongholicus Bunge. [Fabaceae; astragali radix], Salvia miltiorrhiza Bunge. [Lamiaceae; radix salviae miltiorrhizae], Ligustrum lucidum W.T.Aiton [Oleaceae; cera chinensis], leeche [not botanical drug], scorpion [not botanical drug] |
| Zhao Y et al. (2018) | Liuwei Dihuang Pills | pill | Wanxi pharmaceutical Co. Ltd., | No | Rehmannia glutinosa (Gaertn.) DC. [Orobanchaceae; radix rehmanniae praeparata], Dioscorea oppositifolia L. [Dioscoreaceae; dioscoreae rhizoma], Cornus officinalis Siebold & Zucc. [Cornaceae; corni fructus], Poria cocos (Schw. ) Wolf. [Polyporaceae; Poria], Alisma plantago-aquatica subsp. orientale (Sam.) Sam. [Alismataceae; alismatis rhizoma], Paeonia × suffruticosa Andrews [Paeoniaceae; moutan cortex] |
| Yu XT et al. (2018) | JTTZ | granule | Jiangyin Tianjiang Pharmaceutical Co. Ltd. (Jiangsu, China) | Yes | Salvia miltiorrhiza Bunge. [Lamiaceae; radix salviae miltiorrhizae], Schisandra chinensis (Turcz.) Baill. [Schisandraceae; schisandrae chinensis fructus], Coptis chinensis Franch. [Ranunculaceae; coptidis rhizoma], Aloe vera (L.) Burm.f. [Asphodelaceae; aloe barbadensis], Anemarrhena asphodeloides Bunge [Asparagaceae; anemarrhenae rhizoma], red yeast rice [not botanical drug], Momordica charantia L. [Cucurbitaceae; fructus momordicae], Zingiber officinale Roscoe [Zingiberaceae; extractum zingiberis liquidum] |
| Xiao Q et al. (2018) | tongxinluo capsules | capsule | Shijiazhuang Yiling pharmaceutical Limited by Share Ltd. | No | Paeonia lactiflora Pall. [Paeoniaceae; paeoniae radix alba], Panax ginseng C.A.Mey. [Araliaceae; folium ginseng], Scolopendra [not botanical drug], Dryobalanops aromatica C.F.Gaertn. [Dipterocarpaceae; bomeolum], leech [not botanical drug], scorpion [not botanical drug], periostracum cicada [not botanical drug] |
| Tong XL et al. (2018) | AMC | granule | Jiangyin Tianjiang Pharmaceutical Co., Ltd. | Yes | Salvia miltiorrhiza Bunge. [Lamiaceae; radix salviae miltiorrhizae], Schisandra chinensis (Turcz.) Baill. [Schisandraceae; schisandrae chinensis fructus], Coptis chinensis Franch. [Ranunculaceae; coptidis rhizoma], Aloe vera (L.) Burm.f. [Asphodelaceae; aloe barbadensis], Anemarrhena asphodeloides Bunge [Asparagaceae; anemarrhenae rhizoma], red yeast rice [not botanical drug], Momordica charantia L. [Cucurbitaceae; fructus momordicae], Zingiber officinale Roscoe [Zingiberaceae; extractum zingiberis liquidum] |
| Tian JX. (2018) | Jinlida | granule | Not Stated | No | Not Stated |
| Tang ZM et al. (2018) | Sancailianmei Particle | partical | Sichuan new green pharmaceutical development holding company | No | Neolitsea cassia (L.) Kosterm. [Lauraceae; cinnamomi cortex], Rehmannia glutinosa (Gaertn.) DC. [Orobanchaceae; radix rehmanniae praeparata], Panax ginseng C.A.Mey. [Araliaceae; folium ginseng], Coptis chinensis Franch. [Ranunculaceae; coptidis rhizoma], Prunus mume (Siebold) Siebold & Zucc. [Rosaceae; fructus mume] |
| Liu J et al. (2018) | Tangzhiqing Tablet | tablet | Shandong Buchang Shenzhou Pharmaceutical Co., Ltd. | No | Paeonia lactiflora Pall. [Paeoniaceae; paeoniae radix alba], Morus alba L. [Moraceae; mori fructus], Nelumbo nucifera Gaertn. [Nelumbonaceae; folium nelumbinis], Salvia miltiorrhiza Bunge. [Lamiaceae; radix salviae miltiorrhizae], Crataegus pinnatifida Bunge [Rosaceae; crataegi fructus] |
| Zhao Y. (2016) | Liuwei Dihuang Pills | pill | Wanxi Pharmaceutical Co., Ltd. | No | Rehmannia glutinosa (Gaertn.) DC. [Orobanchaceae; radix rehmanniae praeparata], Dioscorea oppositifolia L. [Dioscoreaceae; dioscoreae rhizoma], Cornus officinalis Siebold & Zucc. [Cornaceae; corni fructus], Poria cocos (Schw. ) Wolf. [Polyporaceae; Poria], Alisma plantago-aquatica subsp. orientale (Sam.) Sam. [Alismataceae; alismatis rhizoma], Paeonia × suffruticosa Andrews [Paeoniaceae; moutan cortex] |
| Yang X et al. (2016) | Tangshen Formula | granule | Not Stated | No | Rheum officinale Baill. [Polygonaceae; radix et rhizoma rhei], Astragalus mongholicus Bunge. [Fabaceae; astragali radix], Rehmannia glutinosa (Gaertn.) DC. [Orobanchaceae; radix rehmanniae praeparata], Cornus officinalis Siebold & Zucc. [Cornaceae; corni fructus], Panax notoginseng (Burkill) F.H.Chen [Araliaceae; notoginseng radix et rhizoma], Euonymus alatus (Thunb.) Siebold [Celastraceae; euonymi lignum suberalatum], Citrus × aurantium L. [Rutaceae; aurantii fructus immaturus] |
| Xiang L et al. (2016) | Qidan Dihuang Grain | grain | Not Stated | No | Astragalus mongholicus Bunge. [Fabaceae; astragali radix], Salvia miltiorrhiza Bunge. [Lamiaceae; radix salviae miltiorrhizae], Glycyrrhiza glabra L. [Fabaceae; glycyrrhizae radix et rhizoma], Rehmannia glutinosa (Gaertn.) DC. [Orobanchaceae; radix rehmanniae praeparata], Dioscorea oppositifolia L. [Dioscoreaceae; dioscoreae rhizoma] |
| Wu SQ et al. (2016) | Qiwei Baizhu powder/Zhibai Dihuang decoction/combination of Liuwei Dihuang decoction and kidney-Qi pills | powder/decoction/pill | Not Stated | No | Not Stated |
| Hu Y. (2016) | JianYuTangKang | tablet | science and technology center at PLA general hospital, Beijing, China | Yes | Anemarrhena asphodeloides Bunge [Asparagaceae; anemarrhenae rhizoma], Euonymus alatus (Thunb.) Siebold [Celastraceae; euonymi lignum suberalatum], Eleutherococcus senticosus (Rupr. & Maxim.) Maxim. [Araliaceae; eleutherococci senticosi rhizoma] |
| Hu Y et al. (2016) | JianYuTangKang | tablet | Science and Technology Center, Chinese PLA General Hospital, Beijing, China | Yes | Anemarrhena asphodeloides Bunge [Asparagaceae; anemarrhenae rhizoma], Euonymus alatus (Thunb.) Siebold [Celastraceae; euonymi lignum suberalatum], Eleutherococcus senticosus (Rupr. & Maxim.) Maxim. [Araliaceae; eleutherococci senticosi rhizoma] |
| Guo Q et al. (2016) | Sancai powder | powder | Sichuan New Green Pharmaceutical Development Holdings Ltd (Chengdu, China). | No | Neolitsea cassia (L.) Kosterm. [Lauraceae; cinnamomi cortex], Rehmannia glutinosa (Gaertn.) DC. [Orobanchaceae; radix rehmanniae praeparata], Panax ginseng C.A.Mey. [Araliaceae; folium ginseng], Coptis chinensis Franch. [Ranunculaceae; coptidis rhizoma], Prunus mume (Siebold) Siebold & Zucc. [Rosaceae; fructus mume], Asparagus cochinchinensis (Lour.) Merr. [Asparagaceae; asparagi radix] |
| Chui SH et al. (2015) | PSP-1 | granule | Hong Yee Pharmaceutical Factory, Taipa, Macau, China. | No | Citrus × aurantium f. deliciosa (Ten.) M.Hiroe [Rutaceae; citri exocarpium rubrum], Coix lacryma-jobi var. ma-yuen (Rom.Caill.) Stapf [Poaceae; coicis semen], Paederia scandens (Lour.) Merr. [Rubiaceae; P. scandens], pork [not botanical drug] |
| Zhang XX. (2015) | Shen-Qi-Formula | formula | Tianjin Central Pharmaceutical Group Corporation Ltd | No | Rheum officinale Baill. [Polygonaceae; radix et rhizoma rhei], Astragalus mongholicus Bunge. [Fabaceae; astragali radix], Salvia miltiorrhiza Bunge. [Lamiaceae; radix salviae miltiorrhizae], Rehmannia glutinosa (Gaertn.) DC. [Orobanchaceae; radix rehmanniae praeparata], Dioscorea oppositifolia L. [Dioscoreaceae; dioscoreae rhizoma], Cornus officinalis Siebold & Zucc. [Cornaceae; corni fructus], Trichosanthes kirilowii Maxim. [Cucurbitaceae; trichosanthis semen tostum], Panax ginseng C.A.Mey. [Araliaceae; folium ginseng] |
| Xu J et al. (2014) | Gegen Qinlian Decoction (GQD) | decoction | Beijing Shuangqiaoyanjing Chinese herb manufacturer | Yes | Glycyrrhiza glabra L. [Fabaceae; glycyrrhizae radix et rhizoma], Pueraria montana var. lobata (Willd.) Maesen & S.M.Almeida ex Sanjappa & Predeep [Fabaceae; radix puerariae lobatae], Coptis chinensis Franch. [Ranunculaceae; coptidis rhizoma], Scutellaria baicalensis Georgi [Lamiaceae; scutellariae baicalensis radix] |
| Shi G. (2015) | Qi-boosting and Yin-nourishing decoction | decoction | Not Stated | No | Astragalus mongholicus Bunge. [Fabaceae; astragali radix], Ophiopogon japonicus (Thunb.) Ker Gawl. [Asparagaceae; ophiopogonis radix], Paeonia lactiflora Pall. [Paeoniaceae; paeoniae radix alba], Angelica sinensis (Oliv.) Diels [Apiaceae; angelicae sinensis radix], Rehmannia glutinosa (Gaertn.) DC. [Orobanchaceae; radix rehmanniae praeparata], Atractylodes lancea (Thunb.) DC. [Asteraceae; atractylodis lanceae rhizoma pulveratum], Anemarrhena asphodeloides Bunge [Asparagaceae; anemarrhenae rhizoma], Scrophularia ningpoensis Hemsl. [Scrophulariaceae; scrophulariae radix] |
| Qiang G et al. (2015) | Sancaijiangtang powders | powder | Pharmacy Department of the Teaching Hospital of Chengdu, University of Traditional Chinese Medicine (Sichuan, China) | No | Neolitsea cassia (L.) Kosterm. [Lauraceae; cinnamomi cortex], Rehmannia glutinosa (Gaertn.) DC. [Orobanchaceae; radix rehmanniae praeparata], Panax ginseng C.A.Mey. [Araliaceae; folium ginseng], Coptis chinensis Franch. [Ranunculaceae; coptidis rhizoma], Prunus mume (Siebold) Siebold & Zucc. [Rosaceae; fructus mume], Asparagus cochinchinensis (Lour.) Merr. [Asparagaceae; asparagi radix] |
| Luo D et al. (2015) | Compound Danshen Dripping Pill | pill | Tasly Pharmaceutical Group Co., Ltd. (Tianjin, China) | No | Panax notoginseng (Burkill) F.H.Chen [Araliaceae; notoginseng radix et rhizoma], Dryobalanops aromatica C.F.Gaertn. [Dipterocarpaceae; bomeolum], Salvia miltiorrhiza Bunge. [Lamiaceae; radix salviae miltiorrhizae] |
| Liu H. (2015） | ʻSpleen‑kidney‑careʼ Yiqi Huayu and Jiangzhuo decoction | decoction | Not Stated | No | Rheum officinale Baill. [Polygonaceae; radix et rhizoma rhei], Astragalus mongholicus Bunge. [Fabaceae; astragali radix], Salvia miltiorrhiza Bunge. [Lamiaceae; radix salviae miltiorrhizae], Angelica sinensis (Oliv.) Diels [Apiaceae; angelicae sinensis radix], Rehmannia glutinosa (Gaertn.) DC. [Orobanchaceae; radix rehmanniae praeparata], Dioscorea oppositifolia L. [Dioscoreaceae; dioscoreae rhizoma], Cornus officinalis Siebold & Zucc. [Cornaceae; corni fructus], Curcuma longa L. [Zingiberaceae; curcumae longae rhizoma], Alisma plantago-aquatica subsp. orientale (Sam.) Sam. [Alismataceae; alismatis rhizoma], Atractylodes macrocephala Koidz. [Asteraceae; atractylodis rhizoma pulveratum], Poria cocos (Schw. ) Wolf. [Polyporaceae; Poria], Coix lacryma-jobi var. ma-yuen (Rom.Caill.) Stapf [Poaceae; coicis semen], leech [not botanical drug], Cullen corylifolium (L.) Medik. [Fabaceae; psoraleae fructus], Rosa rugosa Thunb. [Rosaceae; fructus rosae], Codonopsis pilosula (Franch.) Nannf. [Campanulaceae; codonopsis radix], Inula helenium L. [Asteraceae; elecampane] |
| Lian FM, Tian JX et al. (2015) | Jinlida | granule | Shijiazhuang Yiling Pharmaceutical Co. (Shijiazhuang, China) | Yes | Salvia miltiorrhiza Bunge. [Lamiaceae; radix salviae miltiorrhizae], Ophiopogon japonicus (Thunb.) Ker Gawl. [Asparagaceae; ophiopogonis radix], Rehmannia glutinosa (Gaertn.) DC. [Orobanchaceae; radix rehmanniae praeparata], Cornus officinalis Siebold & Zucc. [Cornaceae; corni fructus], Panax ginseng C.A.Mey. [Araliaceae; folium ginseng], Atractylodes macrocephala Koidz. [Asteraceae; atractylodis rhizoma pulveratum], Poria cocos (Schw. ) Wolf. [Polyporaceae; Poria], Pueraria montana var. lobata (Willd.) Maesen & S.M.Almeida ex Sanjappa & Predeep [Fabaceae; radix puerariae lobatae], Coptis chinensis Franch. [Ranunculaceae; coptidis rhizoma], Anemarrhena asphodeloides Bunge [Asparagaceae; anemarrhenae rhizoma], Polygonatum sibiricum Redouté [Asparagaceae; solomonseal rhizome], Sophora flavescens Aiton [Fabaceae; sophorae flavescentis radix], Reynoutria multiflora (Thunb.) Moldenke [Polygonaceae; caulis polygoni multiflori], Eupatorium fortunei Turcz. [Asteraceae; eupatorii herba], Epimedium sagittatum (Siebold & Zucc.) Maxim. [Berberidaceae; epimedii folium], Litchi chinensis Sonn. [Sapindaceae; litchi semen], Lycium barbarum L. [Solanaceae; cortex lycii radicis] |
| Lian FM, Wu L et al. (2015) | Compound Danshen Dripping Pill | pill | Tasly Pharmaceutical Group Co.,Tianjin, China | No | Panax notoginseng (Burkill) F.H.Chen [Araliaceae; notoginseng radix et rhizoma], Dryobalanops aromatica C.F.Gaertn. [Dipterocarpaceae; bomeolum], Salvia miltiorrhiza Bunge. [Lamiaceae; radix salviae miltiorrhizae] |
| Li P et al. (2015) | Tangshen Formula | granule | Jiangyin Tianjiang Pharmaceutical, Jiangsu, China | Yes | Rheum officinale Baill. [Polygonaceae; radix et rhizoma rhei], Astragalus mongholicus Bunge. [Fabaceae; astragali radix], Rehmannia glutinosa (Gaertn.) DC. [Orobanchaceae; radix rehmanniae praeparata], Cornus officinalis Siebold & Zucc. [Cornaceae; corni fructus], Panax notoginseng (Burkill) F.H.Chen [Araliaceae; notoginseng radix et rhizoma], Euonymus alatus (Thunb.) Siebold [Celastraceae; euonymi lignum suberalatum], Citrus × aurantium L. [Rutaceae; aurantii fructus immaturus] |
| Zhao H et al. (2014) | oral Chinese decoctions according to their syndromes diagnosed with TCM standards | decoction | Hospital Affiliated to Chengdu TCM University | No | oral Chinese decoctions according to their syndromes diagnosed with TCM standards |
| Watanabe K et al. (2014) | Goshajinkigan | powder | Tsumura Co., Tokyo, Japan | Yes | Neolitsea cassia (L.) Kosterm. [Lauraceae; cinnamomi cortex], Rehmannia glutinosa (Gaertn.) DC. [Orobanchaceae; radix rehmanniae praeparata], Dioscorea oppositifolia L. [Dioscoreaceae; dioscoreae rhizoma], Cornus officinalis Siebold & Zucc. [Cornaceae; corni fructus], Alisma plantago-aquatica subsp. orientale (Sam.) Sam. [Alismataceae; alismatis rhizoma], Poria cocos (Schw. ) Wolf. [Polyporaceae; Poria], Paeonia × suffruticosa Andrews [Paeoniaceae; moutan cortex], Achyranthes bidentata Blume [Amaranthaceae; achyranthis radix], Plantago asiatica L. [Plantaginaceae; plantaginis semen], Aconitum carmichaelii Debeaux [Ranunculaceae; aconiti radix] |
| Ko CH et al. (2014) | NF3, comprised of Astragali Radix and Radix Rehmanniae | granule | herbal shops in mainland China in Shanxi Province and Hebei Province, | Yes | Astragalus mongholicus Bunge. [Fabaceae; astragali radix], Rehmannia glutinosa (Gaertn.) DC. [Orobanchaceae; radix rehmanniae praeparata] |
| Tu X et al. (2013) | Fructus Mume Formula | decoction | Sichuan Neautus Traditional Chinese Medicine, Inc., Ltd. | Yes | Neolitsea cassia (L.) Kosterm. [Lauraceae; cinnamomi cortex], Angelica sinensis (Oliv.) Diels [Apiaceae; angelicae sinensis radix], Asarum heterotropoides F.Schmidt [Aristolochiaceae; asari radix et rhizoma], Phellodendron amurense Rupr. [Rutaceae; phellodendri cortex pulveratus], Panax ginseng C.A.Mey. [Araliaceae; folium ginseng], Coptis chinensis Franch. [Ranunculaceae; coptidis rhizoma], Zingiber officinale Roscoe [Zingiberaceae; extractum zingiberis liquidum], Prunus mume (Siebold) Siebold & Zucc. [Rosaceae; fructus mume], Aconitum carmichaelii Debeaux [Ranunculaceae; aconiti radix], Zanthoxylum bungeanum Maxim. [Rutaceae; zanthoxyli pericarpium] |
| Tsai C et al. (2013) | modified Hungqi Guizhi Wuwu Tang (MHGWT) | powder | Kaiser Pharmaceuticals Co., Ltd.,Tainan, Taiwan. | Yes | Astragalus mongholicus Bunge. [Fabaceae; astragali radix], Neolitsea cassia (L.) Kosterm. [Lauraceae; cinnamomi cortex], Paeonia lactiflora Pall. [Paeoniaceae; paeoniae radix alba], Zingiber officinale Roscoe [Zingiberaceae; rhizoma zingiberis praeparatum], Ziziphus jujuba Mill. [Rhamnaceae; fructus jujubae], Spatholobus suberectus Dunn [Fabaceae; caulis spatholobi], Pheretima aspergillum [not botanical drug] |
| Tong XL et al. (2013) | TM81 (or Tang-Min-Ling-Wan) | capsule | Tasly Pharmaceutical, Tianjin, China | No | Rheum officinale Baill. [Polygonaceae; radix et rhizoma rhei], Paeonia lactiflora Pall. [Paeoniaceae; paeoniae radix alba], Citrus × aurantium f. deliciosa (Ten.) M.Hiroe [Rutaceae; citri exocarpium rubrum], Coptis chinensis Franch. [Ranunculaceae; coptidis rhizoma], Scutellaria baicalensis Georgi [Lamiaceae; scutellariae baicalensis radix] |
| Ma JW et al. (2013) | zishentongluo (ZSTL) | granule | Shenzhen Sanjiu Pharmaceutical Company, Shenzhen, China | No | Astragalus mongholicus Bunge. [Fabaceae; astragali radix], Carthamus tinctorius L. [Asteraceae; carthami flos], Angelica sinensis (Oliv.) Diels [Apiaceae; angelicae sinensis radix], Rehmannia glutinosa (Gaertn.) DC. [Orobanchaceae; radix rehmanniae praeparata], Cornus officinalis Siebold & Zucc. [Cornaceae; corni fructus], Schisandra chinensis (Turcz.) Baill. [Schisandraceae; schisandrae chinensis fructus], Poria cocos (Schw. ) Wolf. [Polyporaceae; Poria], Epimedium sagittatum (Siebold & Zucc.) Maxim. [Berberidaceae; epimedii folium], Pheretima aspergillum [not botanical drug], Curcuma aromatica Salisb. [Zingiberaceae; curcumae radix], Cuscuta chinensis Lam. [Convolvulaceae; cuscutae semen] |
| Ji LN et al. (2013) | Xiaoke Pill | pill | Guangzhou Zhongyi Pharmaceutical, Guangzhou, China | No | Astragalus mongholicus Bunge. [Fabaceae; astragali radix], Zea mays L. [Poaceae; amylum maydis], Rehmannia glutinosa (Gaertn.) DC. [Orobanchaceae; radix rehmanniae praeparata], Dioscorea oppositifolia L. [Dioscoreaceae; dioscoreae rhizoma], Trichosanthes kirilowii Maxim. [Cucurbitaceae; trichosanthis semen tostum], Schisandra chinensis (Turcz.) Baill. [Schisandraceae; schisandrae chinensis fructus], Pueraria montana var. lobata (Willd.) Maesen & S.M.Almeida ex Sanjappa & Predeep [Fabaceae; radix puerariae lobatae], |
| Grant S et al. (2013) | Jiangtang Xiaozhi capsules | capsule | Tianjin Zhongxin Pharmaceutical Group Corporation Ltd | No | Astragalus mongholicus Bunge. [Fabaceae; astragali radix], Curcuma longa L. [Zingiberaceae; curcumae longae rhizoma], Coptis chinensis Franch. [Ranunculaceae; coptidis rhizoma], Ligustrum lucidum W.T.Aiton [Oleaceae; cera chinensis], Litchi chinensis Sonn. [Sapindaceae; litchi semen], Ecklonia kurome OKAM.[Alariaceae; kelp] |
| Fang ZH et al. (2013) | Danzhijiangtang capsule (DJC) | capsule | Pharmaceutical Formulations Centre, First Affiliated Hospital of Anhui College of Traditional Chinese Medicine | No | Not Stated |
| Leung,P. (2012) | combined herbal formula | granule | Not Stated | No | Astragalus mongholicus Bunge. [Fabaceae; astragali radix], Rehmannia glutinosa (Gaertn.) DC. [Orobanchaceae; radix rehmanniae praeparata], Dioscorea oppositifolia L. [Dioscoreaceae; dioscoreae rhizoma], Cornus officinalis Siebold & Zucc. [Cornaceae; corni fructus], Alisma plantago-aquatica subsp. orientale (Sam.) Sam. [Alismataceae; alismatis rhizoma], Schisandra chinensis (Turcz.) Baill. [Schisandraceae; schisandrae chinensis fructus], Atractylodes macrocephala Koidz. [Asteraceae; atractylodis rhizoma pulveratum], Poria cocos (Schw. ) Wolf. [Polyporaceae; Poria], Smilax glabra Roxb. [Smilacaceae; smilacis glabrae rhizoma], Paeonia × suffruticosa Andrews [Paeoniaceae; moutan cortex], Reynoutria multiflora (Thunb.) Moldenke [Polygonaceae; caulis polygoni multiflori], Stephania tetrandra S.Moore [Menispermaceae; stephaniae tetrandrae radix] |
| Chen DS et al. (2012) | modified Ling-Gui-Zhu-Gan decoction | decoction | Not Stated | No | Rheum officinale Baill. [Polygonaceae; radix et rhizoma rhei], Glycyrrhiza glabra L. [Fabaceae; glycyrrhizae radix et rhizoma], Neolitsea cassia (L.) Kosterm. [Lauraceae; cinnamomi cortex], Atractylodes macrocephala Koidz. [Asteraceae; atractylodis rhizoma pulveratum], Poria cocos (Schw. ) Wolf. [Polyporaceae; Poria], Codonopsis pilosula (Franch.) Nannf. [Campanulaceae; codonopsis radix] |
| Li S et al. (2011) | Tangzu Yuyang Ointment (TYO) | ointment | Key Laboratory of Chemistry for Natural Products of Guizhou Province and Chinese Academy of Science | Yes | Rheum officinale Baill. [Polygonaceae; radix et rhizoma rhei], Angelica sinensis (Oliv.) Diels [Apiaceae; angelicae sinensis radix], Phellodendron amurense Rupr. [Rutaceae; phellodendri cortex pulveratus], Atractylodes lancea (Thunb.) DC. [Asteraceae; atractylodis lanceae rhizoma pulveratum], Panax notoginseng (Burkill) F.H.Chen [Araliaceae; notoginseng radix et rhizoma], Dryobalanops aromatica C.F.Gaertn. [Dipterocarpaceae; bomeolum], Coptis chinensis Franch. [Ranunculaceae; coptidis rhizoma], Conioselinum anthriscoides 'Chuanxiong' [Apiaceae; cnidii rhizoma], Arnebia euchroma (Royle ex Benth.) I.M.Johnst. [Boraginaceae; arnebiae radix], Calamus draco Willd. [Arecaceae; draconis sanguis], Gypsum fibrosum praeparatum [not botanical drug] |
| Li FL et al. (2011) | Shengji Powder | powder | Pharmacy Department of Yueyang Hospital | No | Gypsum fibrosum praeparatum [not botanical drug], Calamus draco Willd. [Arecaceae; draconis sanguis], Boswellia sacra Flück. [Burseraceae], Commiphora myrrha (T.Nees) Engl. [Burseraceae; gummi myrrha], Dryobalanops aromatica C.F.Gaertn. [Dipterocarpaceae; bomeolum] |
| You WH et al. (2009) | modified Danggui Sini decoction | decoction | Not Stated | No | Salvia miltiorrhiza Bunge. [Lamiaceae; radix salviae miltiorrhizae], Neolitsea cassia (L.) Kosterm. [Lauraceae; cinnamomi cortex], Paeonia lactiflora Pall. [Paeoniaceae; paeoniae radix alba], Angelica sinensis (Oliv.) Diels [Apiaceae; angelicae sinensis radix], Asarum heterotropoides F.Schmidt [Aristolochiaceae; asari radix et rhizoma], Cornus officinalis Siebold & Zucc. [Cornaceae; corni fructus], Atractylodes macrocephala Koidz. [Asteraceae; atractylodis rhizoma pulveratum], Poria cocos (Schw. ) Wolf. [Polyporaceae; Poria], Spatholobus suberectus Dunn [Fabaceae; caulis spatholobi], Pheretima aspergillum [not botanical drug], Pseudostellaria heterophylla (Miq.) Pax [Caryophyllaceae; pseudostellariae radix] |
| Luo XX et al. (2009) | Qiming Granule | granule | Zhejiang Wanma Pharmaceutical Ltd., China | No | Astragalus mongholicus Bunge. [Fabaceae; astragali radix], Lycium barbarum L. [Solanaceae; lycii fructus], Pueraria montana var. lobata (Willd.) Maesen & S.M.Almeida ex Sanjappa & Predeep [Fabaceae; radix puerariae lobatae], Rehmannia glutinosa (Gaertn.) DC. [Orobanchaceae; radix rehmanniae praeparata] |
| Chao ML er al. (2009) | Traditional Chinese Medicine | tablet | Long Shun Rong Pharmaceutical Inc (Tianjin, China) | No | Lonicera japonica Thunb. [Caprifoliaceae; caulis lonicerae japonicae], Astragalus mongholicus Bunge. [Fabaceae; astragali radix], Coptis chinensis Franch. [Ranunculaceae; coptidis rhizoma] |
| Zhang YC. (2008) | shengmai injection | injection | Shanghai First Pharmaceutical Plant of Chinese Herbal Medicine | No | Panax ginseng C.A.Mey. [Araliaceae; folium ginseng], Schisandra chinensis (Turcz.) Baill. [Schisandraceae; schisandrae chinensis fructus], Ophiopogon japonicus (Thunb.) Ker Gawl. [Asparagaceae; ophiopogonis radix] |
| Lueng P. (2008） | herbal formulation | granule | a Shanghai hospital | No | Astragalus mongholicus Bunge. [Fabaceae; astragali radix], Rehmannia glutinosa (Gaertn.) DC. [Orobanchaceae; radix rehmanniae praeparata], Dioscorea oppositifolia L. [Dioscoreaceae; dioscoreae rhizoma], Cornus officinalis Siebold & Zucc. [Cornaceae; corni fructus], Alisma plantago-aquatica subsp. orientale (Sam.) Sam. [Alismataceae; alismatis rhizoma], Schisandra chinensis (Turcz.) Baill. [Schisandraceae; schisandrae chinensis fructus], Atractylodes macrocephala Koidz. [Asteraceae; atractylodis rhizoma pulveratum], Poria cocos (Schw. ) Wolf. [Polyporaceae; Poria], Smilax glabra Roxb. [Smilacaceae; smilacis glabrae rhizoma], Paeonia × suffruticosa Andrews [Paeoniaceae; moutan cortex], Reynoutria multiflora (Thunb.) Moldenke [Polygonaceae; caulis polygoni multiflori], Stephania tetrandra S.Moore [Menispermaceae; stephaniae tetrandrae radix] |
| Wei YQ. (2007) | Weidong Kang decoction | decoction | Department of Pharmacy, Chongqing Institute of Traditional Chinese Medicine | No | Rheum officinale Baill. [Polygonaceae; radix et rhizoma rhei], Magnolia officinalis Rehder & E.H.Wilson [Magnoliaceae; flos magnoliae officinalis], Atractylodes macrocephala Koidz. [Asteraceae; atractylodis rhizoma pulveratum], Citrus × aurantium L. [Rutaceae; aurantii fructus immaturus], Areca catechu L. [Arecaceae; arecae catechi semen], Bupleurum chinense DC. [Apiaceae; bupleuri radix], Dendrobium nobile Lindl. [Orchidaceae; caulis dendrobii] |
| Xue J. (2006） | Kaixin Capsule | capsule | pharmaceutical department of the First Affiliated Hospital of Guangzhou University of TCM | No | Carthamus tinctorius L. [Asteraceae; carthami flos], Ophiopogon japonicus (Thunb.) Ker Gawl. [Asparagaceae; ophiopogonis radix], Atractylodes lancea (Thunb.) DC. [Asteraceae; atractylodis lanceae rhizoma pulveratum], Crataegus pinnatifida Bunge [Rosaceae; crataegi fructus], Conioselinum anthriscoides 'Chuanxiong' [Apiaceae; cnidii rhizoma], Panax quinquefolius L. [Araliaceae; american ginseng], Cyperus rotundus L. [Cyperaceae; cyperi rhizoma], Typha angustifolia L. [Typhaceae; pollen typhae], trogopterus dung [not botanical drug] |
| Chen CB. (2005) | Jiang Zhuo Mixture | decoction | Not Stated | No | Astragalus mongholicus Bunge. [Fabaceae; astragali radix], Salvia miltiorrhiza Bunge. [Lamiaceae; radix salviae miltiorrhizae], Dioscorea oppositifolia L. [Dioscoreaceae; dioscoreae rhizoma], Atractylodes lancea (Thunb.) DC. [Asteraceae; atractylodis lanceae rhizoma pulveratum], Pueraria montana var. lobata (Willd.) Maesen & S.M.Almeida ex Sanjappa & Predeep [Fabaceae; radix puerariae lobatae], Lablab purpureus subsp. purpureus [Fabaceae; semen lablab album], Coix lacryma-jobi var. ma-yuen (Rom.Caill.) Stapf [Poaceae; coicis semen], Crataegus pinnatifida Bunge [Rosaceae; crataegi fructus], Hordeum vulgare L. [Poaceae; fructus hordei germinatus], Endothelium Corneum Ggigeriae Galli [not botanical drug] |
| Wang Q. (2005) | prescription for lowering glucose and lipid | tea | Not Stated | No | Astragalus mongholicus Bunge. [Fabaceae; astragali radix], Salvia miltiorrhiza Bunge. [Lamiaceae; radix salviae miltiorrhizae], Carthamus tinctorius L. [Asteraceae; carthami flos], Lycium barbarum L. [Solanaceae; lycii fructus], Prunus persica (L.) Batsch [Rosaceae; persicae semen], Paeonia × suffruticosa Andrews [Paeoniaceae; moutan cortex], leech [not botanical drug], Crataegus pinnatifida Bunge [Rosaceae; crataegi fructus], Polygonatum sibiricum Redouté [Asparagaceae; solomonseal rhizome], Reynoutria multiflora (Thunb.) Moldenke [Polygonaceae; caulis polygoni multiflori], Panax quinquefolius L. [Araliaceae; american ginseng] |
| Huang M. (2004) | yitangning granule | granule | Hangzhou Municipal Hospital of Traditional Chinese Medicine | No | Lycium barbarum L. [Solanaceae; lycii fructus], Angelica sinensis (Oliv.) Diels [Apiaceae; angelicae sinensis radix], Rehmannia glutinosa (Gaertn.) DC. [Orobanchaceae; radix rehmanniae praeparata], Dioscorea oppositifolia L. [Dioscoreaceae; dioscoreae rhizoma], Cornus officinalis Siebold & Zucc. [Cornaceae; corni fructus], Cuscuta chinensis Lam. [Convolvulaceae; cuscutae semen], Conioselinum anthriscoides 'Chuanxiong' [Apiaceae; cnidii rhizoma], Conioselinum anthriscoides 'Chuanxiong' [Apiaceae; cnidii rhizoma] |
| Nagaki Y et al. (2003) | Goshajinkigan (Niu-che-shen-qi-wan in Chinese) | granule | Tsumura & Co. Ltd., Tokyo, Japan | No | Neolitsea cassia (L.) Kosterm. [Lauraceae; cinnamomi cortex], Rehmannia glutinosa (Gaertn.) DC. [Orobanchaceae; radix rehmanniae praeparata], Dioscorea oppositifolia L. [Dioscoreaceae; dioscoreae rhizoma], Cornus officinalis Siebold & Zucc. [Cornaceae; corni fructus], Alisma plantago-aquatica subsp. orientale (Sam.) Sam. [Alismataceae; alismatis rhizoma], Poria cocos (Schw. ) Wolf. [Polyporaceae; Poria], Paeonia × suffruticosa Andrews [Paeoniaceae; moutan cortex], Achyranthes bidentata Blume [Amaranthaceae; achyranthis radix], Plantago asiatica L. [Plantaginaceae; plantaginis semen], Aconitum carmichaelii Debeaux [Ranunculaceae; aconiti radix] |
| Wu ST. (2000) | Bai Fu Ling decoction | decoction | Not Stated | No | Panax ginseng C.A.Mey. [Araliaceae; folium ginseng], Trichosanthes kirilowii Maxim. [Cucurbitaceae; trichosanthis semen tostum], Magnetite [not botanical drug], Rehmannia glutinosa (Gaertn.) DC. [Orobanchaceae; radix rehmanniae praeparata], Poria cocos (Schw. ) Wolf. [Polyporaceae; Poria], Coptis chinensis Franch. [Ranunculaceae; coptidis rhizoma], Scrophularia ningpoensis Hemsl. [Scrophulariaceae; scrophulariae radix], Dendrobium nobile Lindl. [Orchidaceae; caulis dendrobii], Endothelium Corneum Ggigeriae Galli [not botanical drug], Dioscorea collettii var. hypoglauca (Palib.) S.J.Pei & C.T.Ting [Dioscoreaceae; dioscoreae hypoglaucae rhizoma], Rubus chingii Hu [Rosaceae; fructus rubi], Cnidium monnieri (L.) Cusson [Apiaceae; cnidi fructus] |
| Ren HY. (2000) | Tang Zhi Min Capsule | capsule | Not Stated | No | Astragalus mongholicus Bunge. [Fabaceae; astragali radix], Carthamus tinctorius L. [Asteraceae; carthami flos], Lycium barbarum L. [Solanaceae; lycii fructus], Angelica sinensis (Oliv.) Diels [Apiaceae; angelicae sinensis radix], Rehmannia glutinosa (Gaertn.) DC. [Orobanchaceae; radix rehmanniae praeparata], Trichosanthes kirilowii Maxim. [Cucurbitaceae; trichosanthis semen tostum], leech [not botanical drug], Anemarrhena asphodeloides Bunge [Asparagaceae; anemarrhenae rhizoma], Codonopsis pilosula (Franch.) Nannf. [Campanulaceae; codonopsis radix], Spatholobus suberectus Dunn [Fabaceae; caulis spatholobi], Conioselinum anthriscoides 'Chuanxiong' [Apiaceae; cnidii rhizoma], Chaenomeles speciosa (Sweet) Nakai [Rosaceae; chaenomelis fructus], Taxillus chinensis (DC.) Danser [Loranthaceae; herba taxilli] |
| Li R. (2000) | TCM recipe | decoction | Not Stated | No | Pueraria montana var. lobata (Willd.) Maesen & S.M.Almeida ex Sanjappa & Predeep [Fabaceae; radix puerariae lobatae], Salvia miltiorrhiza Bunge. [Lamiaceae; radix salviae miltiorrhizae], Conioselinum anthriscoides 'Chuanxiong' [Apiaceae; cnidii rhizoma]，Rehmannia glutinosa (Gaertn.) DC. [Orobanchaceae; radix rehmanniae praeparata], Dioscorea oppositifolia L. [Dioscoreaceae; dioscoreae rhizoma], Cornus officinalis Siebold & Zucc. [Cornaceae; corni fructus], Poria cocos (Schw. ) Wolf. [Polyporaceae; Poria], Alisma plantago-aquatica subsp. orientale (Sam.) Sam. [Alismataceae; alismatis rhizoma], Paeonia × suffruticosa Andrews [Paeoniaceae; moutan cortex] |
| Li M. (1999) | Reinforced Capsule of Gastrodia and Eucommia | capsule | Guizhou Hongyu Pharmaceutical Co., Ltd. | No | Gastrodia elata Blume [Orchidaceae; gastrodiae rhizoma], Eucommia ulmoides Oliv. [Eucommiaceae; cortex eucommiae] |
| Gao Y et al. (1998) | Tang Shen Ning | liquid | Not Stated | No | Rheum officinale Baill. [Polygonaceae; radix et rhizoma rhei], Astragalus mongholicus Bunge. [Fabaceae; astragali radix], Salvia miltiorrhiza Bunge. [Lamiaceae; radix salviae miltiorrhizae], Rehmannia glutinosa (Gaertn.) DC. [Orobanchaceae; radix rehmanniae praeparata], Cornus officinalis Siebold & Zucc. [Cornaceae; corni fructus], Alisma plantago-aquatica subsp. orientale (Sam.) Sam. [Alismataceae; alismatis rhizoma], leech [not botanical drug], Conioselinum anthriscoides 'Chuanxiong' [Apiaceae; cnidii rhizoma], Pseudostellaria heterophylla (Miq.) Pax [Caryophyllaceae; pseudostellariae radix], Euryale ferox Salisb. [Nymphaeaceae; euryales semen], Rosa laevigata Michx. [Rosaceae; fructus rosae laevigatae] |
| Wang XB. (1997) | Tangshenkang Capsule | capsule | The First Affiliated Hospital of Xinxiang Medical College, Henan | No | Astragalus mongholicus Bunge. [Fabaceae; astragali radix], Salvia miltiorrhiza Bunge. [Lamiaceae; radix salviae miltiorrhizae], Paeonia lactiflora Pall. [Paeoniaceae; paeoniae radix alba], Angelica sinensis (Oliv.) Diels [Apiaceae; angelicae sinensis radix], Prunus persica (L.) Batsch [Rosaceae; persicae semen], Conioselinum anthriscoides 'Chuanxiong' [Apiaceae; cnidii rhizoma], Leonurus japonicus Houtt. [Lamiaceae; herba leonuri] |
| Zhu L. (1992) | Tong Yu Ling tablets | tablet | Not Stated | No | Rheum officinale Baill. [Polygonaceae; radix et rhizoma rhei], Prunus persica (L.) Batsch [Rosaceae; persicae semen], leech [not botanical drug] |
| Hale P et al. (1989) | xiao ke tea | tea | Not Stated | No | Not Stated |
